# Analysis of gastric microbiome reveals three distinctive microbial communities associated with the occurrence of gastric cancer

**DOI:** 10.1186/s12866-022-02594-y

**Published:** 2022-07-23

**Authors:** Dehua Liu, Rutong Zhang, Si Chen, Baolin Sun, Kaiguang Zhang

**Affiliations:** 1 The First Affiliated Hospital of USTC, Division of Life Sciences and Medicine, University of Science and Technology of China, Hefei, Anhui China; 2 School of Life Sciences, University of Science and Technology of China, Hefei, Anhui China

**Keywords:** Microbiota, Bacterial community, Gastric cancer, Predictive model

## Abstract

**Background:**

Gastric microbial dysbiosis were reported to be associated with gastric cancer (GC). This study aimed to explore the variation, diversity, and composition patterns of gastric bacteria in stages of gastric carcinogenesis based on the published datasets.

**Methods:**

We conducted a gastric microbial analysis using 10 public datasets based on 16S rRNA sequencing, including 1270 gastric biopsies of 109 health control, 183 superficial gastritis (SG), 135 atrophic gastritis (AG), 124 intestinal metaplasia (IM), 94 intraepithelial neoplasia (IN), 344 GC, and 281 adjacent normal tissues. And QIIME2-pipeline, DESeq2, NetMoss2, vegan, igraph, and RandomForest were used for the data processing and analysis.

**Results:**

We identified three gastric microbial communities among all the gastric tissues. The first community (designate as GT-H) was featured by the high abundance of *Helicobacter*. The other two microbial communities, namely GT-F, and GT-P, were featured by the enrichment of phylum Firmicutes and Proteobacteria, respectively. The distribution of GC-associated bacteria, such as *Fusobacterium*, *Peptostreptococcus*, *Streptococcus*, and *Veillonella* were enriched in tumor tissues, and mainly distributed in GT-F type microbial communities. Compared with SG, AG, and IM, the bacterial diversity in GC was significantly reduced. And the strength of microbial interaction networks was initially increased in IM but gradually decreased from IN to GC. In addition, Randomforest models constructed in in GT-H and GT-F microbial communities showed excellent performance in distinguishing GC from SG and precancerous stages, with varied donated bacteria.

**Conclusions:**

This study identified three types of gastric microbiome with different patterns of composition which helps to clarify the potential key bacteria in the development of gastric carcinogenesis.

**Supplementary Information:**

The online version contains supplementary material available at 10.1186/s12866-022-02594-y.

## Introduction

Gastric cancer (GC) is the fifth most common cancer globally, which is a health threat worldwide [1]. Generally, advanced age, male sex, family history, high salt diet, atrophic gastritis, and *Helicobacter pylori* infection were reported as risk factors of GC [2]. In recent years, many studies based on next-generation sequencing technologies have revealed a close relationship between gastric bacteria and GC, suggesting that non-*H. pylori* bacteria may be associated with the progression of GC [3].

GC tissues were reported to have a unique micro-ecology, in which microbial diversity and *Helicobacter* abundance were reduced, and other genera such as *Actinobacteria*, *Lactobacillus*, *Clostridium,* etc. were enriched [4]. Whereas another study reported that *Lactococcus*, *Veillonella*, *Fusobacterium*, and *Leptotrichia* were enriched in patients with GC, compared to patients with functional dyspepsia [5]. Coker et al. identified that *Dialister pneumosintes*, *Parvimonas micra*, *Slackia exigua*, *Peptostreptococcus stomatis*, and *Streptococcus anginosus* were centralities in the ecological network of GC and also inhabit the oral cavity [6].

Several studies identified that some gastric bacteria were also associated with gastric precancerous lesions [7–9]. *Rhizobiales* was found to be more enriched in patients with intestinal metaplasia (IM) than in those with superficial gastritis (SG) [10]. Sung et al. have identified that *Granulicatella*, *Actinomyces*, *Rothia*, *Peptostreptococcus*, *Streptococcus*, *Abiotrophia*, and *Parvimonas* are associated with atrophic gastritis (AG) or IM in patients following *H. pylori* successful eradication [11]. A recent study reported that *Sphingomonas* and *Aquincola tertiaricabonis* were increased in IM, Neisseriaceae, *Streptococcus*, and *Haemophilus parainfluencae* were significantly enriched in patients with intraepithelial neoplasia (IN), and *Veillonella* and *Lactobacillus* were more abundant in GC [7].

However, these gastric microbiota associate studies did not reach consistent conclusions about the bacteria related to GC, which may be influenced by the effect of age and geographic location of participants, extraction and sequencing methods of samples, and differential analysis and statistical methods. In addition, the high abundance of *H. pylori* in the stomach of some patients with positive *H. pylori* infection could significantly affect the microbial diversity and composition structure of gastric microbiome [9, 12–14]. We suggested that specific microbial composition patterns influenced by some abundant taxon could affect the identification of disease-related microbes. As previous studies have demonstrated that the gut microbiome can be classified into distinct enterotypes, which help us understand the bacteria associated with human health and disease [15]. Whereas the compositional patterns of gastric microbiota and its association with the occurrence of GC were not identified yet.

In this work, we investigated several 16S sequencing datasets from gastric microbiota-related studies on the key bacteria at different stages of GC progression, using multiple methods with batch effect removed. And we explored the composition patterns of gastric microbiome and further analysed the changes in GC-related bacteria, microbial diversity and ecology based on three identified gastric microbial communities, as well as the value of GC prediction.

## Materials and methods

### Datasets collection

We conducted a gastric microbial study using datasets from 10 publications that included 16S sequencing data for one or more biopsies of gastritis, precancerous lesions, GC, and carcinoma adjacent tissues. The datasets were labelled as d1 [16], d2 [5], d3 [7], d4 [17], d5 [18], d6 [6], d7 [19], d8 [20], d9 [21], and d10 [9]. These studies had similar exclusion criteria, such as subjects not taking proton pump inhibitors, prebiotics, and antibiotics at least a month before sample collection. And the raw sequence data were retrieved from the Sequence Read Archive of the NCBI database.

### Data processing

Sequencing quality filtering and analysis of different dataset were performed using the QIIME2 pipeline separately (v2020.11)[22]. Raw reads were filtered and dereplicated using VSEARCH and Deblur plugin (Paired-end reads were merged before quality control), which generated the feature table and feature representative sequences. The samples with a total abundance (total number of sequences obtained from the sample) > 3000, features with a total abundance > 10 and observed in at least two samples were reserved for subsequent analysis. The SILVA 16S database (v138) was used for taxonomy assignment of sequence datasets and performed by the QIIME2 plugin feature classifier [23]. Each dataset was processed independently to gain the count tables, with features ranging from phylum to genus. The abundant tables were merged at each taxonomy level and converted into relative abundance tables for microbial community analysis, bacterial composition analysis, and random forest model construction. The feature abundance tables were rarefied to the sequencing depth of 3000 to filter the very low abundance sequences or dataset-specific taxa for taxonomic discovery analysis, ecology analysis, and diversity analysis of gastric microbiota.

### Taxonomy discovery analysis

Taxonomic discovery analysis was performed using the R package DESeq2 based on genera (relative abundance > 0.1%), with cohort information and relative abundance of *Helicobacter* added to adjust age, gender, *H. pylori*, and batch effects [24]. We further utilized R package NetMoss2 [25], which based on Sparcc [6] network modules and showed good performance in removing batch effects, to identify robust GC-associated bacteria from multiple datasets.

### Microbial composition pattern and cluster analysis

The microbial composition pattern and cluster analysis to identify gastric microbial community was performed based on genera (relative abundance > 0.1%) using Jensen-Shannon divergence (JSD) distance and the Partitioning Around Medoids (PAM) clustering algorithm and visualized using between-class analysis (BCA) and principal coordinates analysis (PCoA), as previous study reported [26].

### Alpha diversity and beta diversity analysis

Alpha diversity analysis with Shannon, Chao1, and Simpson indexes was performed based on rarefied abundance table at genus level using Picante package. Beta diversity with Bray–Curtis distance matrices was performed based on rarefied abundance table at genus level using Vegan package.

### Microbial interaction network analysis

The identification of co-occurring and co-exclusion bacteria based on rarefied abundance table at the genus level with relative abundance > 0.1% was estimated using the SparCC algorithm [6], and visualized by Gephi (v0.9.2) [27].

### Machine-learning approach for model building

All the random forest models were conducted based on genera with relative abundance > 0.1% using R package RandomForest. The combined dataset for GC and other diagnosis group was randomly split into training and test sets in a ratio of 7:3. Further, we used R package Boruta to select important bacteria that contributed significantly to the classification and constructed the models based on these selected genera. The receiver operating characteristic (ROC) analysis was performed to illustrate the performances of classification models using R package ROCR.

### Statistical analysis

The differences in Alpha diversity were assessed using an ANOVA test for multiple groups. Multiple group comparisons of dissimilarities were performed using the permutational multivariate analysis of variance test (PERMANOVA). Network parameters including topological coefficient, closeness, and betweenness were estimated using igraph (v1.2.5) and compared using the Wilcoxon test. Data visualizations were performed by the R Project (v4.1.0). All *p* values < 0.05 after multiple comparisons correction using false discovery rate method were considered significantly different.

## Results

### Data demographics and assessment

We collected most of the current published gastric microbial sequencing studies related to the progression of GC. These studies are mainly in Asia, especially in China (Supplementary Fig. 1A). The datasets presented pertain to a total of 1270 gastric mucosal samples after exclusion. And these samples were classified into health control (HC, 109), superficial gastritis (SG, 183), atrophic gastritis (AG, 135), intestinal metaplasia (IM, 124), intraepithelial neoplasia (IN, 94), gastric cancer (GC, 344), and carcinoma adjacent normal tissues (CAN, 281) groups, according to histological diagnosis (Supplementary Fig. 1B). The baseline characteristics of metadata were presented in Supplementary Table 1 and Supplementary Table 2. The significance age and gender differences among the groups were adjusted using DESeq2 in subsequent differential analysis.

### Key bacteria in GC

**Fig. 1:**
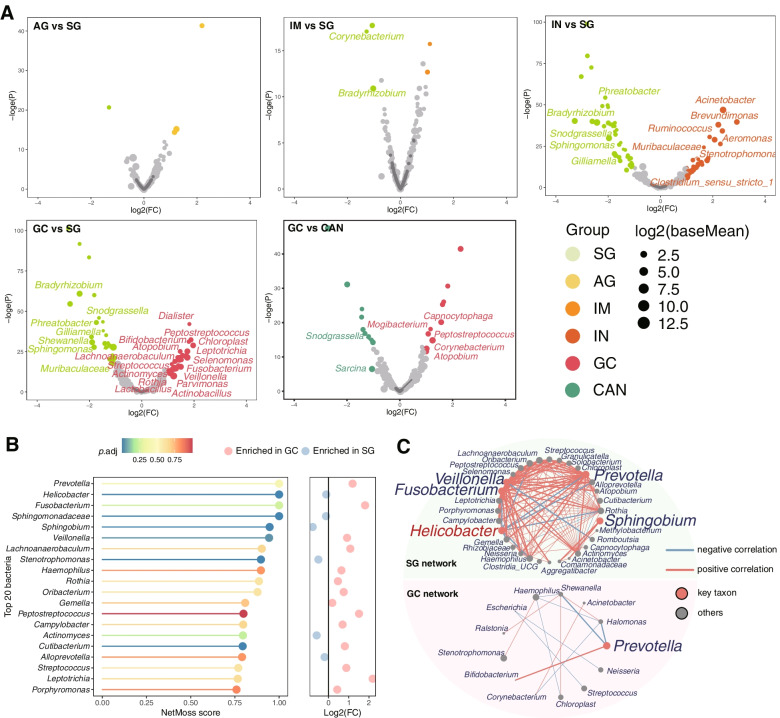
**A** DESeq2 identifies specific bacterial taxa associated with the development of GC, with age, gender, H. pylori, and batch effects adjusted. **B** NetMoss2 identifies specific bacterial taxa in microbial Sparcc networks between GC and SG. **C** Sparcc networks between GC and SG constructed by NetMoss2. HC: health control, SG: superficial gastritis, AG: atrophic gastritis, IM: intestinal metaplasia, IN: intraepithelial neoplasia, GC: gastric cancer, CAN: carcinoma adjacent normal tissues. Log2(FC): Log2(Fold Change), -loge(P): the negative of log base e of *P* value

We revealed that enriched *Acinetobacter*, *Fusobacterium*, *Lactococcus*, *Lactobacillus*, *Peptostreptococcus*, *Prevotella*, *Streptococcus*, *Selenomonas*, and *Veillonella* in GC were found in more than one study (Supplementary Fig. 2 and Supplementary Table 3) [5–7, 19, 20]. We then assessed the genera enriched in each diagnosis group in combined datasets using DESeq2 (Fig. 1A). Comparing with SG group, *Acinetobacter* and *Ruminococcus* were enriched in IN group, *Actinobacillus*, *Actinomyces*, *Bifidobacterium*, *Fusobacterium*, *Lactobacillus*, *Parvimonas*, *Rothia*, *Streptococcus*, *Veillonella*, etc. were enriched in GC group. In addition, significantly enriched *Peptostreptococcus* and *Atopobium*, and depleted *Snodgrassella* were observed in GC group compared with both CAN and SG groups. *Gilliamella*, *Sphingomonas*, *Bradyrhizobium*, and *Phreatobacter* were depleted in both IN and GC groups compared with SG group. We further utilized R package NetMoss2 based on microbial Sparcc network of GC and SG to identify GC-related bacteria. The results also showed that *Fusobacterium*, *Peptostreptococcus*, *Streptococcus*, and *Veillonella* were enriched in GC tissues (Fig. 1B). *Prevotella* was the key taxon in GC and SG networks, and positive correlated with *Fusobacterium*, *Streptococcus*, and *Peptostreptococcus* in microbial network of SG biopsies (Fig. 1C).

### Three microbial communities are defined in gastric microbiome

**Fig. 2:**
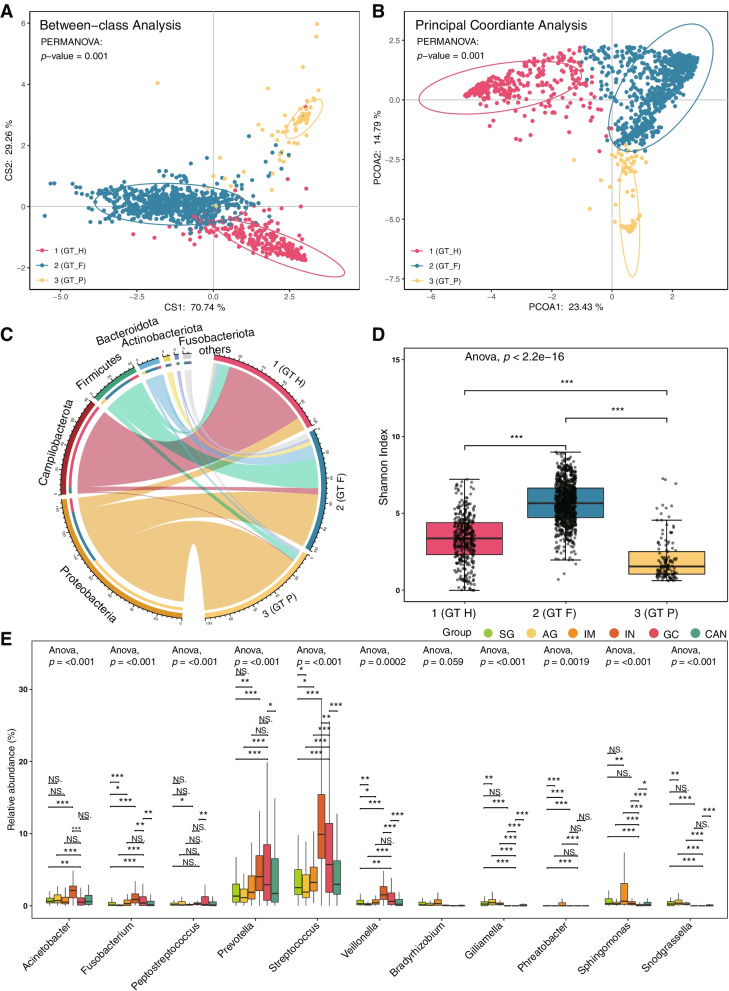
Differences of microbial composition and diversity between three bacterial communities. **A**, **B** The multidimensional cluster analysis of the gastric microbiome at the genus level shows three distinct clusters of samples. **C** The abundance of the gastric microbiota is shown for phylum level in three microbial communities. **D** Alpha diversity was estimated by the Shannon index at the genus level for three microbial communities. **E** The relative abundance of significantly changed bacteria in GT-F type samples among different disease groups. GT: gastric type. *: *p*.adj < 0.05, **: *p*.adj < 0.01, ***: *p*.adj < 0.001

We performed a multidimensional cluster analysis using JSD distance and PAM algorithm to evaluate the bacterial community of the gastric microbiome and revealed that the samples formed three distinct clusters (Fig. 2A and Fig. 2B, Supplementary Table 4). The first cluster was identifiable by the variation in the levels of phylum Campilobacterota (mean relative abundance = 74.18%, *p* < 0.001), as well as high abundant *Helicobacter*, which we designated as GT-H (Gastric type H, Fig. 2C, Supplementary Fig. 3, and Supplementary Fig. 4). The second cluster was named GT-F since the proportion of phylum Firmicutes increased (mean relative abundance = 26.30%, *p* < 0.001). In the biopsies of third cluster (GT-P), the bacteria belonging to phylum Proteobacteria (mean relative abundance = 88.86%, *p* < 0.001) were dominant. Most GC-enriched genera, such as *Fusobacterium*, *Peptostreptococcus, Streptococcus*, *Veillonella*, etc. were mainly distributed in samples of GT-F (Supplementary Fig. 4). *Helicobacter* was most enriched in GT-H group, and *Pseudomonas* was more abundant in GT-P group. the Shannon index of alpha diversity analysis showed that GT-F has the highest microbial diversity, followed by GT-H and GT-P (Fig. 2D). In biopsies of GT-H and GT-F, we also observed that *Fusobacterium*, *Prevotella*, *Streptococcus*, and *Veillonella* were increased in IN and GC groups, whereas *Gilliamella* and *Snodgrassella* were reduced (Fig. 2E and Supplementary Fig. 5). The enriched *Acinetobacter* in GC group and *Pseudomonas* in CAN group were observed in GT-P type samples (Supplementary Fig. 6).

In addition, Samples of GT-H and GT-F were not limited to the disease and projects, each existed in multiple diagnosis groups and datasets (Supplementary Fig. 7). Whereas most samples of GT-P belong to GC and CAN, and belong to dataset d8.

### Gastric microbial diversity is altered in three gastric microbial communities

**Fig. 3:**
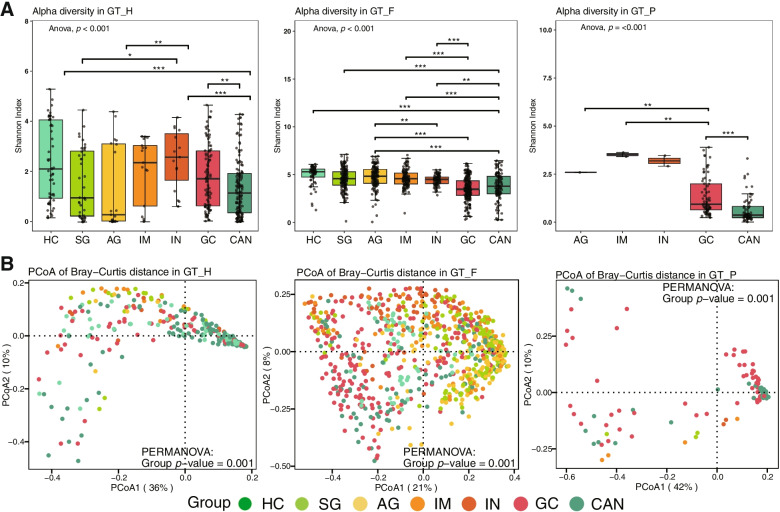
Gastric microbial diversity and community structure at the genus level among disease groups. **A** Alpha diversity was estimated by Shannon diversity index for disease groups. **B** PCoA plots and PERMANOVA test based on Bray–Curtis distance for disease groups. HC: health control, SG: superficial gastritis, AG: atrophic gastritis, IM: intestinal metaplasia, IN: intraepithelial neoplasia, GC: gastric cancer, CAN: carcinoma adjacent normal tissues. *: adjusted *p* < 0.05, **: adjusted *p* < 0.01, ***: adjusted *p* < 0.001

We further performed the alpha diversity analysis with Shannon, Chao1, and Simpson indexes between diagnosis groups (Fig. 3A and Supplementary Fig. 8). In GT-H, the diversity of gastric microbiota was significantly increased in GC group compared to CAN groups. In GT-F, a decreasing trend of diversity, richness, and evenness of gastric microbiota was found across AG to GC. We also observed that the microbial diversity of GC was significantly higher than CAN in samples of GT-P. Differences in microbial community structure were further evaluated in three gastric microbial types (Fig. 3B). The beta diversity using Bray–Curtis distance matrices showed that the composition of mucosal microbiota had significant differences between diagnosis groups. To verify these results, we also performed the analysis of alpha and beta diversity between disease groups in independent datasets (Supplementary Figs. 9, 10, 11, 12, 13 and 14). The significantly decreased alpha diversity of GC microbiome in both GT-H and GT-F communities only appeared in dataset d3. Whereas the variety of beta diversity between different diagnosis groups was observed in multiple datasets of different microbial communities.

### Gastric microbiota ecology is altered in three gastric microbial communities

**Fig. 4:**
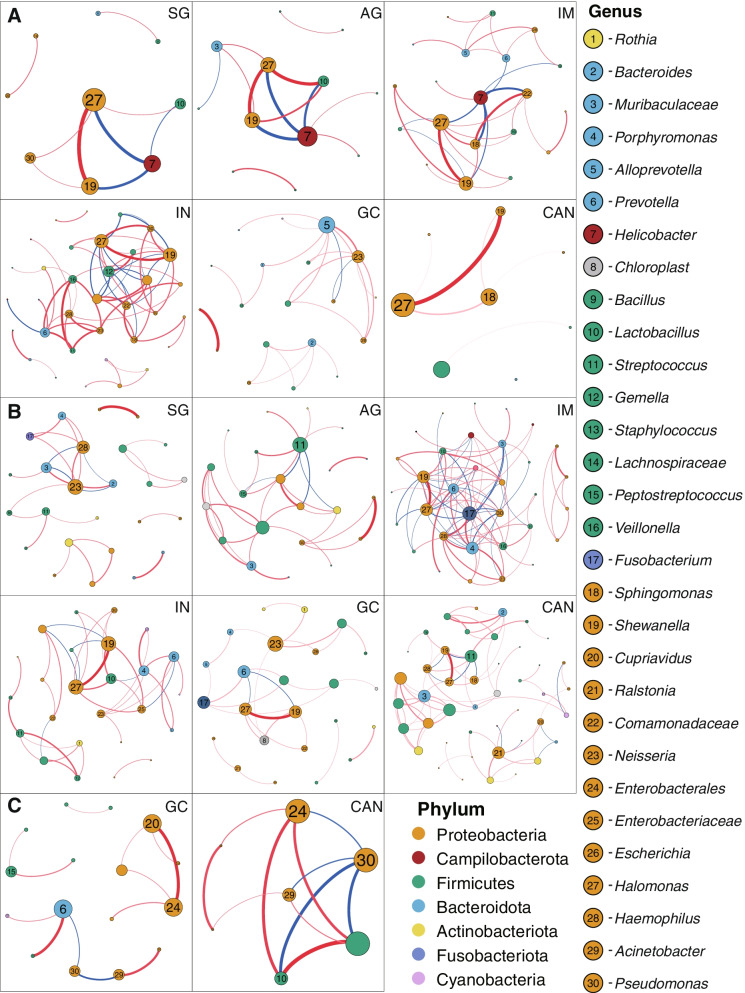
Correlation networks of the gastrointestinal genus among disease groups. **A** The interactions of bacteria in gastric biopsies in samples of GT-H. **B** The interactions of bacteria in samples of GT-F. **C** The interactions of bacteria in samples of GT-P. The size of nodes corresponds to weighted node connectivity scores, and the nodes were colored by phylum. Red edges denote positive correlations and blue edges denote negative correlations. The interactions of bacteria in GC and CAN groups were exhibited with strengths > 0.4, and in SG, AG, IM, IN groups with strengths > 0.6. HC: health control, SG: superficial gastritis, AG: atrophic gastritis, IM: intestinal metaplasia, IN: intraepithelial neoplasia, GC: gastric cancer, CAN: carcinoma adjacent normal tissues

We further performed a microbial co-occurrence and co-exclusion network and topology analysis at different stages of GC progression to explore the interaction of gastric microbiota. In GT-H group, the strength of co-occurring interactions among genera increased in AG, IM, IN, and decreased in GC and CAN (Fig. 4A and Fig. 4B, Supplementary Table 5 and Supplementary Table 6). Co-exclusion interactions were observed from SG to IM tissues, with *Helicobacter* as the interaction node. In GT-F type samples, the interaction between gastric bacteria in IM group was the strongest, and the interaction strength was gradually weakened from IN to GC group. We observed that *Prevotella*, *Streptococcus*, *Neissera, Shewanella*, *Halomonas,* etc. had a higher degree of centrality and strong co-occurrence interaction with other genera. Whereas *Pseudomonas* co-excluded with several genera in CAN groups of GT-P type microbial community (Fig. 4C).

### The value of gastric microbiota in predicting GC

**Fig. 5:**
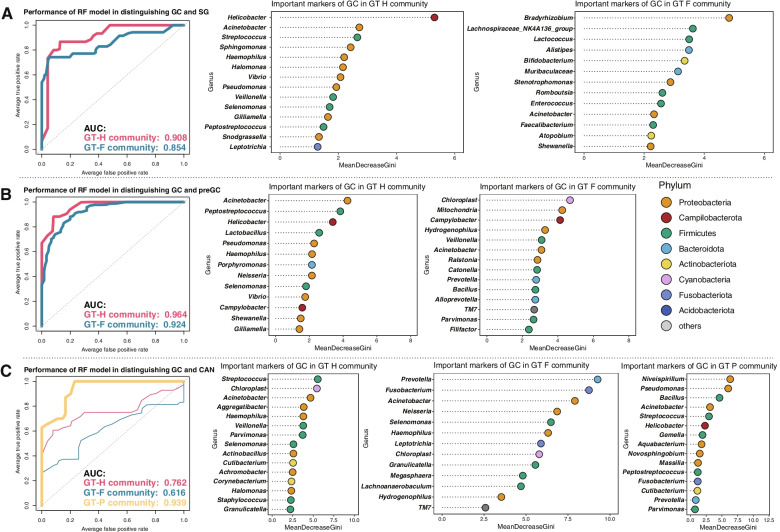
Based on genera selected by Boruta, the performance of gastric microbial models in prediction GC was analysed by receiver operating characteristic (ROC) curve analysis. **A** The discriminatory potential of microbial model in distinguishing GC and SG. **B** The discriminatory potential of microbial model in distinguishing GC and precancerous lesions, including AG, IM, and IN. **C** The discriminatory potential of microbial model in distinguishing GC and CAN. SG: superficial gastritis, IM: intestinal metaplasia, GC: gastric cancer, CAN: carcinoma adjacent normal tissues, preGC: precancerous lesions of gastric cancer. AUC: area under the curve

We further constructed random forest models based on the genera with relative abundance > 0.1% in different gastric microbial communities, to assess the value and general applicability of gastric microbiota in predicting GC. The improtant genera that contributed to the models were selected by Boruta. The models in GT-H and GT-F microbial communities performed well in distinguishing GC and SG, with area under the curve (AUC) of 0.908 and 0.854 (Fig. 5A). Meanwhile, the models constructed for distinguishing GC and precancerous lesions, including AG, IM, and IN, also showed excellent performance with AUC of 0.964 and 0.924 (Fig. 5B). *Haemophilus* and *Selenomonas* were the main contributers in models distinguishing GC from others groups of GT-H microbial community, and *Acinetobacter* was important in models of GT-F type community. In addition, the bacterial model showed excellent performance in distinguishing GC and CAN with an AUC of 0.939 in samples of GT-P (Fig. 5C). The genera that contributed significantly to the gastric microbial models were also showed in Supplementary Table 7.

### The influence factors affect gastric microbiota

Finally, we analysed the influence of factors, such as age, gender, geolocation, sequencing region, and batch effects on these GC-associated bacteria. The results showed that *Prevotella*, *Lactobacillus*, and *Streptococcus* were more abundant in patients with advanced age, *Halomonas* and *Shewanella* were enriched in female subjects (Supplementary Fig. 15 and Supplementary Fig. 16). *Halomonas* and *Shewanella* were also found more abundant in participants of North China, whereas *Pseudomonas* was most enriched in participants of Liaoning, China (Supplementary Fig. 17). In addition, *Halomonas* and *Shewanella* were more abundant in datasets with V3-V4 region sequenced than those with V4 region sequenced. It was more likely to detect that the higher relative abundance of gastric dominant bacteria in the V3-V4 and V4 region sequencing datasets (Supplementary Fig. 18 and Supplementary Fig. 19). Whereas the distribution of these genera in other sequencing datasets was significantly affected by *H. pylori* infection.

## Discussion

In this study, we investigated several previous gastric microbial studies, reanalysed the associated datasets to identify the microbial community and key bacteria, which contribute to the occurrence and progression of GC. We revealed three types of gastric microbial communities, which influenced microbial diversity, interaction, and predictive value among different diagnosis groups.

We furthermore identified *Fusobacterium*, *Peptostreptococcus*, *Streptococcus*, and *Veillonella* that were associated with the development of GC in multiple datasets. Some of these GC-related bacteria were also reported in several previous publications, but without consistent conclusions. Wang et al. found that a taxon of *Veillonella* and two taxa of *Lactobacillus* were enriched in GC using Linear discriminant analysis Effect Size analysis [7]. And a previous study revealed more GC-enriched bacterial taxa, including *Peptostreptococcus*, *Streptococcus anginosus*, *Slackia*, *Gemella*, and *Fusobacterium *[6].

Enrichment of these GC-related bacteria has also been found in other digestive tumours. *Streptococcus*, *Peptostreptococcus*, *Prevotella*, *Fusobacterium*, *Porphyromonas gingivalis*, and *Capnocytophaga gingivalis* have been identified that correlate strongly with oral cancer [28]. *Fusobacterium nucleatum* could cause opportunistic infections and be associated with colorectal cancer [29]. *Peptostreptococcus anaerobius* could promote colorectal carcinogenesis and modulate tumor immunity [30]. The mechanism of these bacteria in promoting the development of colon cancer has been progressively clarified.

We found that most bacteria associated with GC have been reported that have pro-inflammatory activities and can release virulence factors, suggesting that long-term chronic inflammation and toxins accumulation caused by pathogenic bacteria may be risk factors in the occurrence of GC [31–33]. Such as Group B *Streptococcus*, a leading cause of pneumonia, sepsis, and meningitis, was an asymptomatic member of the gastrointestinal microbiota [34]. *Streptococcus* was significantly increased and dominant in GC tissues, its role in in digestive tract needs further study. In addition, modulation of the NF-κB signaling pathway by infectious bacteria was linked to gastrointestinal cancers initiation and development [35]. NF-κB was highly activated at the site of infection by *H. pylori*, *F. nucleatum*, etc., for its antimicrobial activity and maintenance of tissue homeostasis [36].

We also found that *Acinetobacter* were enriched in IN group. While in the other two studies, *Acinetobacter* was reported more abundant in GC compared with CAN [19, 20]. *Acinetobacter lwoffii* and *Streptococcus anginosus* were enriched in patients with persistent inflammation after *H. pylori* was eradicated [11]. Gastritis and hypergastrinemia were not specific for *H. pylori*, but can be induced by *Acinetobacter lwoffii* capable of infecting the stomach in a mouse model [37]. These results suggested that *Acinetobacter* was associated with the occurrence and development of gastric neoplasia. The genera related to the AG and IM were not consistent in our research and previous studies. However, a study further explored the relationship between gastric microbiota and the occurrence of GC in a mouse model, and found that the bacteria of patients with IM or GC could selectively colonize the stomach of germ-free mice and induce gastric precancerous lesions [38].

Intestinal microbiota could be distinguished into different enterotypes, such as ET B, ET F, and ET P driven by Bacteroides, Firmicutes, and *Prevotella*, respectively [39]. While, high abundance of *H. pylori* in stomach could disrupt the diversity and composition of gastric mucosal microbiome, which might affect the identification of GC related bacteria [40]. Inspired by these findings, we analysed the composition patterns of gastric microbiome and identified three microbial communities, which were defined as GT-H, GT-F, and GT-P. The GT-H was driven by *H. pylori* infection, with a relative abundance of *Helicobacter* exceeding 40% in most samples. GT-F was more like a mixed type of gastric microbiota, with Firmicutes, Proteobacteria, and Bacteroidota in higher proportions. GT-F also had the highest microbial diversity, with most GC-related bacteria, such as *Fusobacterium*, *Prevotella*, *Streptococcus*, *Veillonella*, etc. enriched. GT-P type gastric microbial community was similar to GT-H type, both showing the dominance of a single genus, which might be the result of infection with certain pathogenic bacteria. The dominant *H. pylori* in stomach could cause long-term chronic inflammation of gastric mucosa and lead to the occurrence of AG [41]. Therefore, the more abundant bacteria in GT-P type microbiota might play an important role in the development of GC. However, the formation of such groups could also be caused by environmental contamination of samples.

GT-H and GT-F types were more common in gastric biopsies, and not limited to the disease and projects. Whereas most samples of GT-P were tumour tissues and para-cancer tissues, which were distinguished by an overrepresentation of two genera of Proteobacteria, namely *Acinetobacter* and *Pseudomonas*. In several gastric microbial studies, only Chen et al. reported that *Pseudomonas aeruginosa* was enriched in non-cancerous tissues [20]. Due to the low proportion of GT-P type samples in other datasets, several high abundance bacteria might mask the changes of *Pseudomonas* in GC progression. The diversity and composition of the gastric microbiota were significantly altered in different stomach normal, peritumoral, and tumoral microhabitats [19]. We suggested further dividing gastric microhabitats according to the different microbial communities to study the association between gastric microbiota and gastric carcinogenesis.

The diversity analysis revealed that a significantly decreased bacterial alpha diversity in GC and CAN be compared with AG, IM, and IN in samples of GT-F and GT-P, confirming previous studies [6, 7]. The reduced bacterial interaction strengths were also observed in GC and CAN be compared with other disease groups. The genera *Prevotella, Streptococcus, Shewanella*, and *Halomonas* had a higher degree of centrality and strong co-occurrence interaction with other genera across the development of GC in samples of GT-H and GT-F. We also found that *Helicobacter* was co-excluded with multiple genera in GT-H type tissues with precancerous lesions. It suggested that protecting the diversity of gastric microbiota and stabilizing the microenvironment was a potential therapeutic strategy to reduce the infection rate of *H. pylori* or other pathogens.

*Shewanella* and *Halomonas* were reported to be enriched in the peritumoral microhabitat and non-atrophic chronic gastritis, and were also associated with rectal cancer, which was an opportunistic pathogen associated with gastrointestinal infection [7, 19, 42]. We further identified that *Shewanella* and *Halomonas* were more enriched in female patients and northern populations. *Pseudomonas* was more abundant in Liaoning Province, China, and was mainly concentrated in one dataset. The distribution of these three genera may be affected by the sequencing region, as well*. Prevotella* and *Streptococcus* were more abundant in patients with advanced age. These results suggest that age, sex, geolocation, and sequencing region are important factors affecting the abundance of several gastric bacteria. In addition, the GC-associated genera, such as *Fusobacterium*, *Peptostreptococcus*, *Prevotella*, *Streptococcus*, and *Veillonella* were less affected by batch effects and enriched in most datasets. These potentially pathogenic bacteria enriched in GC may form a harmful microecological network and participate in the pathological process of gastric mucosa.

Several studies had identified that the significantly changed non-*H. pylori* genera could be used as the potential microbial biomarkers for GC and precancerous lesions [6–8]. A study in Linqu, China revealed that the panel of *Helicobacter*, *Bacillus*, *Capnocytophaga*, and *Prevotella* could help to distinct advanced gastric lesions and showed predictive value for lesion progression [43]. However, we found that different performances of the gastric bacterial model to predict GC in different microbial communities with varied contributing genera. The model of microbiota showed an excellent performance in distinguishing GC and CAN in samples of GT-P. Micriobial models construced in GT-H and GT-P communities also showed good perfromance in distinguishing GC from SG, as well as the stages of precancerous lesions. It suggests that the influence of microbial community should be fully considered in studying the predictive value of microbiome to disease.

Nevertheless, our study still has several limitations. We enrolled multiple gastric microbial datasets for analysis of GC-associated bacteria, but each dataset only covered a few stages of GC development. Most of the samples in microbial community GT-P belong to a single data set and the relationship between this special microbial community and the development of GC needs further investigation. In addition, we discussed the effects of age, gender, and geolocation on the gastric microbiota, while the influence of other factors such as diet, smoking, and alcohol consumption still needs to be addressed. We identified the key genera and microbial communities which could have contributed to the development of GC and were less affected by batch effects, but further studies from a wider geographical area using the same sequencing and analysis strategy were needed to confirm these conclusions.

In conclusion, our study demonstrated the changes of gastric microbiota across the development of GC in multiple datasets. We identified three gastric microbial communities, namely GT-H, GT-F, and GT-P, which exhibited different variations in microbial diversity and interactions between different disease groups. There were distinct distributions of GC-associated bacteria, such as *Fusobacterium*, *Peptostreptococcus*, *Streptococcus*, and *Veillonella* in the samples of the three gastric microbial types. Microbial models in three types exhibited distinctive values in the prediction of GC from other diagnosis groups. Our study revealed that the composition patterns of gastric microbiota affected the distribution of GC-related bacteria, which might help to understand the prevention and diagnosis of GC and the use of antibiotics in its anti-infective treatment. However, Subsequent confirmatory experimental studies in a broader population are further needed to identify whether these GC-associated bacteria colonize the gastric mucosa and promote its pathological process.

## Supplementary Information

**Additional file 1.** **Supplementary Figure 1. **Merging of datasets from independent studies. (A) The geolocation, amplicon region, and samples size of datasets used in this study. (B) The distribution of the 10 datasets in diagnosis groups. HC: health control, SG: superficial gastritis, AG: atrophic gastritis, IM: intestinal metaplasia, IN: intraepithelial neoplasia, GC: gastric cancer, CAN: carcinoma adjacent normal tissues. **Supplementary Figure 2.** GC-associated bacteria identified in previous studies. The red letters represent bacteria that have been reported to be associated with gastric cancer in more than one study. **Supplementary Figure 3.** The distribution of the top abundant phylum in three microbiome communities. ANOVA test was used for comparison of bacterial relative abundance differences between multiple gastric microbial types, and multiple comparisons were performed by Tukey test and *p* values were adjusted. *: *p.adj <* 0.05, **: *p.adj <* 0.01, ***: *p.adj<* 0.001. **Supplementary Figure 4.** The distribution of the GC-associated bacteria in three microbiome communities. ANOVA test was used for comparison of bacterial relative abundance differences between multiple gastric microbial types, and multiple comparisons were performed by Tukey test and *p* values were adjusted.** *p.adj<* 0.01,*** *p.adj <* 0.001. **Supplementary Figure 5.** The distribution of the GC-associated bacteria in GT H type samples among different disease groups. ANOVA test was used for comparisonof bacterial relative abundance differences between multiple gastric microbialtypes, and multiple comparisons were performed by Tukey test and *p *values were adjusted. * *p.adj *< 0.05, ** *p.adj*< 0.01,*** *p.adj *< 0.001. **Supplementary Figure 6.** The distribution of the GC-associated bacteria in GT P type samples among different disease groups. Wilcoxon test was used for comparison of bacterial relative abundance differences between GC and CAN. **Supplementary Figure 7.** The distribution of samples with different gastric bacterial communities. (A) The distribution of different microbial type samples in multiple diagnosis groups. (B) The distribution of different microbial type samples in different datasets. (C) The distribution of different microbial type samples in different geolocations. **Supplementary Figure 8.** Gastric microbial diversity at the genus level among disease groups. (A) Alpha diversity was estimated by Chao1 richness diversity index for disease groups. (B) Alpha diversity was estimated by Simpson evenness index for disease groups. The ANOVA test was used for comparison of differences between multiple diagnostic groups. *: *p.adj <* 0.05, **: *p.adj<* 0.01,***: *p.adj <* 0.001. **Supplementary Figure 9.** Gastric microbial α diversity of GT H type bacterial community among disease groups in different datasets.The ANOVA test was used for comparison of differences between multiple diagnostic groups. **Supplementary Figure 10** Gastric microbial α diversity of GT F type bacterial community among disease groups in different datasets.The ANOVA test was used for comparison of differences between multiple diagnostic groups. **Supplementary Figure 11.** Gastric microbial α diversity of GT P type bacterial community among disease groups in different datasets.The ANOVA test was used for comparison of differences between multiple diagnostic groups. **Supplementary Figure 12.** Gastric microbial β diversity of GT H type bacterial community among disease groups in different datasets. The PERMANOVA test was used for comparison of differences between multiple diagnostic groups. **Supplementary Figure 13.** Gastric microbial β diversity of GT F type bacterial community among disease groups in different datasets.The PERMANOVA test was used for comparison of differences between multiple diagnostic groups. **Supplementary Figure 14.** Gastric microbial β diversity of GT P type bacterial community among disease groupsin different datasets.The PERMANOVA test was used forcomparison of differences between multiple diagnostic groups. **Supplementary Figure 15.** The distribution of the GC-associated bacteria in different periods of age. The ANOVA test was used for comparison of differences between multiple groups, and multiple comparisons were performed by Tukey test and *p *values were adjusted. * *p.adj <* 0.05, ** *p.adj<* 0.01,*** *p.adj <* 0.001. **Supplementary Figure 16.** The distribution of the GC-associated bacteria in different sexes. The Wilcoxon test was used for comparison of differences between groups. **Supplementary Figure 17.** The distribution of the GC-associated bacteria in different geographical locations. The ANOVA test was used for comparison of bacterial relative abundance differences between multiple groups. **Supplementary Figure 18.** The distribution of the GC-associated bacteria indatasets with different sequencing region. The ANOVA test was used for comparison of bacterial relative abundance differences between multiple groups. **Supplementary Figure 19.** The distribution of the GC-associated bacteria in different datasets. The ANOVA test was used for comparison of bacterial relative abundance differences between multiple groups. 

**Additional file 2.** **Supplementary Table 1.** BaseLine characteristics of datasets. **Supplementary Table 2.** Metadata of this study. **Supplementary Table 3.** GC-associated bacteria identified in previous studies. **Supplementary Table 4.** Different types of gastric microbial communities in different samples. **Supplementary Table 5.** Topological characteristics of SparCC interaction network in different diagnosis groups. **Supplementary Table 6. **Statistical test of network differences between diagnostic groups. **Supplementary Table 7.** The important genera in random forest models selected by Boruta.

## Data Availability

The datasets analysed during the current study are available in the SRA repository of NCBI, with accession number PRJEB11763 (https://www.ncbi.nlm.nih.gov/bioproject/?term=PRJEB11763), PRJEB21497 (https://www.ncbi.nlm.nih.gov/bioproject/?term=PRJEB21497), PRJEB26931 (https://www.ncbi.nlm.nih.gov/bioproject/?term=PRJEB26931), PRJNA239281 (https://www.ncbi.nlm.nih.gov/bioproject/?term=PRJNA239281), PRJNA310127 (https://www.ncbi.nlm.nih.gov/bioproject/?term=PRJNA310127), PRJNA375772 (https://www.ncbi.nlm.nih.gov/bioproject/?term=PRJNA375772), PRJNA428883 (https://www.ncbi.nlm.nih.gov/bioproject/?term=PRJNA428883), PRJNA532731 (https://www.ncbi.nlm.nih.gov/bioproject/?term=PRJNA532731), PRJNA641258 (https://www.ncbi.nlm.nih.gov/bioproject/?term=PRJNA641258), and PRJNA678413 (https://www.ncbi.nlm.nih.gov/bioproject/?term=PRJNA678413).
