# Supplementary material for: Analysis of gastric microbiome reveals three distinctive microbial communities associated with the occurrence of gastric cancer
Source: BMC Microbiol. 2022 Jul 23;22:184. doi: 10.1186/s12866-022-02594-y (PMC9308235; doi:10.1186/s12866-022-02594-y)
Supplement: Supplementary file 1 — Additional file 1. Supplementary Figure 1. Merging of datasets from independent studies. (A) The geolocation, amplicon region, and samples size of datasets used in this study. (B) The distribution of the 10 datasets in diagnosis groups. HC: health control, SG: superficial gastritis, AG: atrophic gastritis, IM: intestinal metaplasia, IN: intraepithelial neoplasia, GC: gastric cancer, CAN: carcinoma adjacent normal tissues. Supplementary Figure 2. GC-associated bacteria identified in previous studies. The red letters represent bacteria that have been reported to be associated with gastric cancer in more than one study. Supplementary Figure 3. The distribution of the top abundant phylum in three microbiome communities. ANOVA test was used for comparison of bacterial relative abundance differences between multiple gastric microbial types, and multiple comparisons were performed by Tukey test and p values were adjusted. *: p.adj < 0.05, **: p.adj < 0.01, ***: p.adj< 0.001. Supplementary Figure 4. The distribution of the GC-associated bacteria in three microbiome communities. ANOVA test was used for comparison of bacterial relative abundance differences between multiple gastric microbial types, and multiple comparisons were performed by Tukey test and p values were adjusted.** p.adj< 0.01,*** p.adj < 0.001. Supplementary Figure 5. The distribution of the GC-associated bacteria in GT H type samples among different disease groups. ANOVA test was used for comparisonof bacterial relative abundance differences between multiple gastric microbialtypes, and multiple comparisons were performed by Tukey test and p values were adjusted. * p.adj < 0.05, ** p.adj< 0.01,*** p.adj < 0.001. Supplementary Figure 6. The distribution of the GC-associated bacteria in GT P type samples among different disease groups. Wilcoxon test was used for comparison of bacterial relative abundance differences between GC and CAN. Supplementary Figure 7. The distribution of samples with different gastric b [file 12866_2022_2594_MOESM1_ESM.docx]

**Supplementary figures**

**
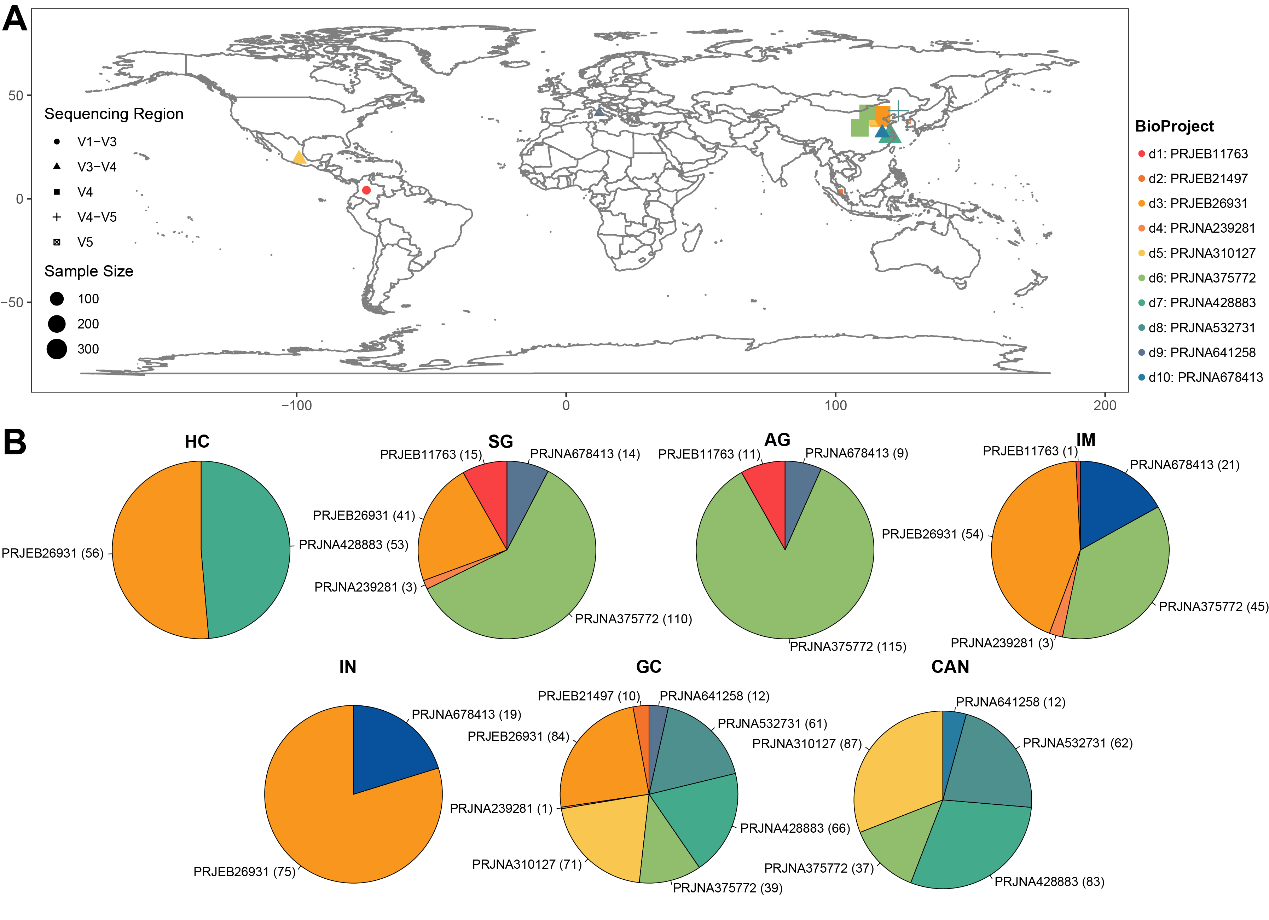
**

**Supplementary figure 1.** Merging of datasets from independent studies. (A) The geolocation, amplicon region, and samples size of datasets used in this study. (B) The distribution of the 10 datasets in diagnosis groups. HC: health control, SG: superficial gastritis, AG: atrophic gastritis, IM: intestinal metaplasia, IN: intraepithelial neoplasia, GC: gastric cancer, CAN: carcinoma adjacent normal tissues.


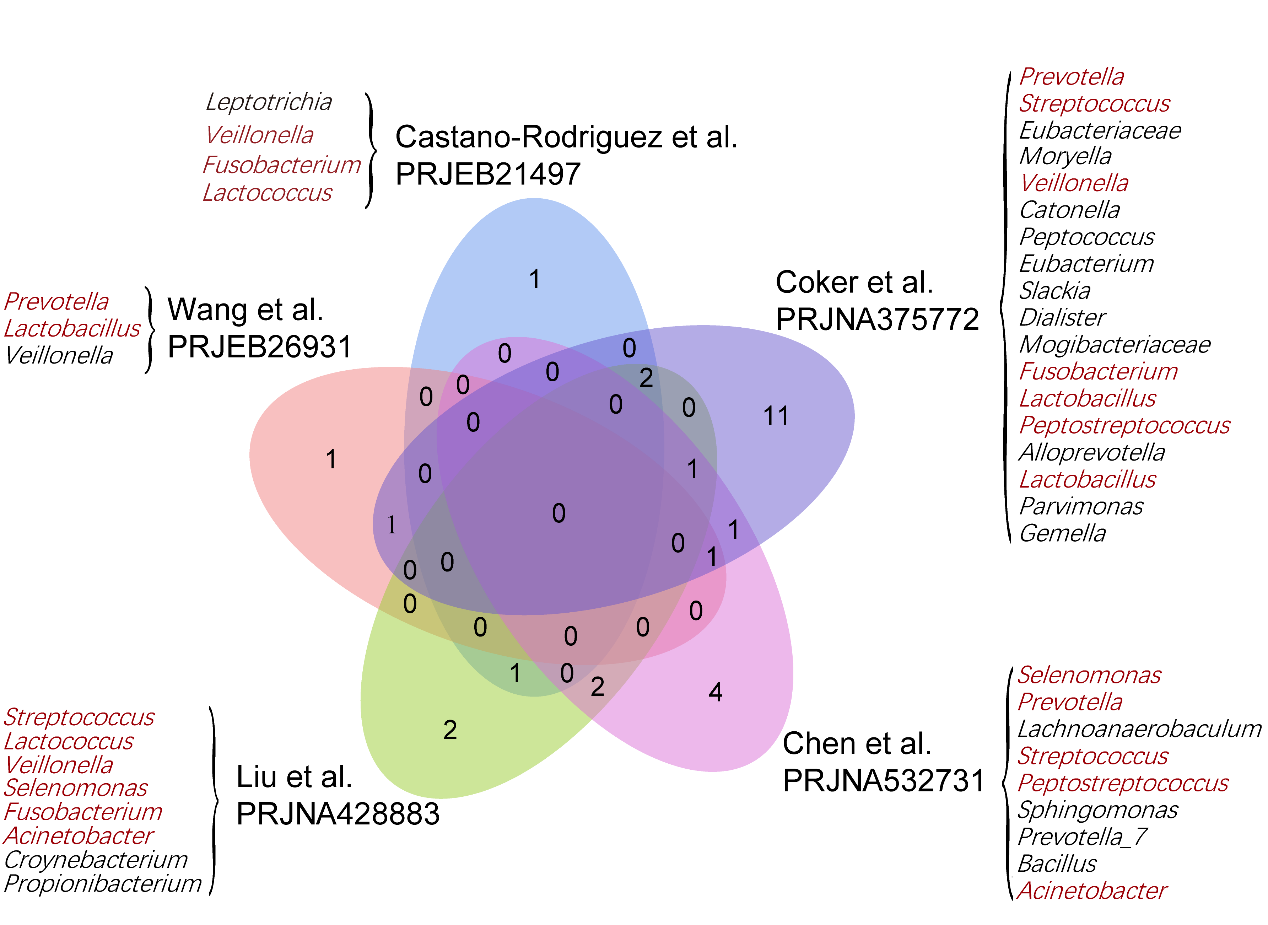


**Supplementary figure 2.** GC-associated bacteria identified in previous studies. The red letters represent bacteria that have been reported to be associated with gastric cancer in more than one study.


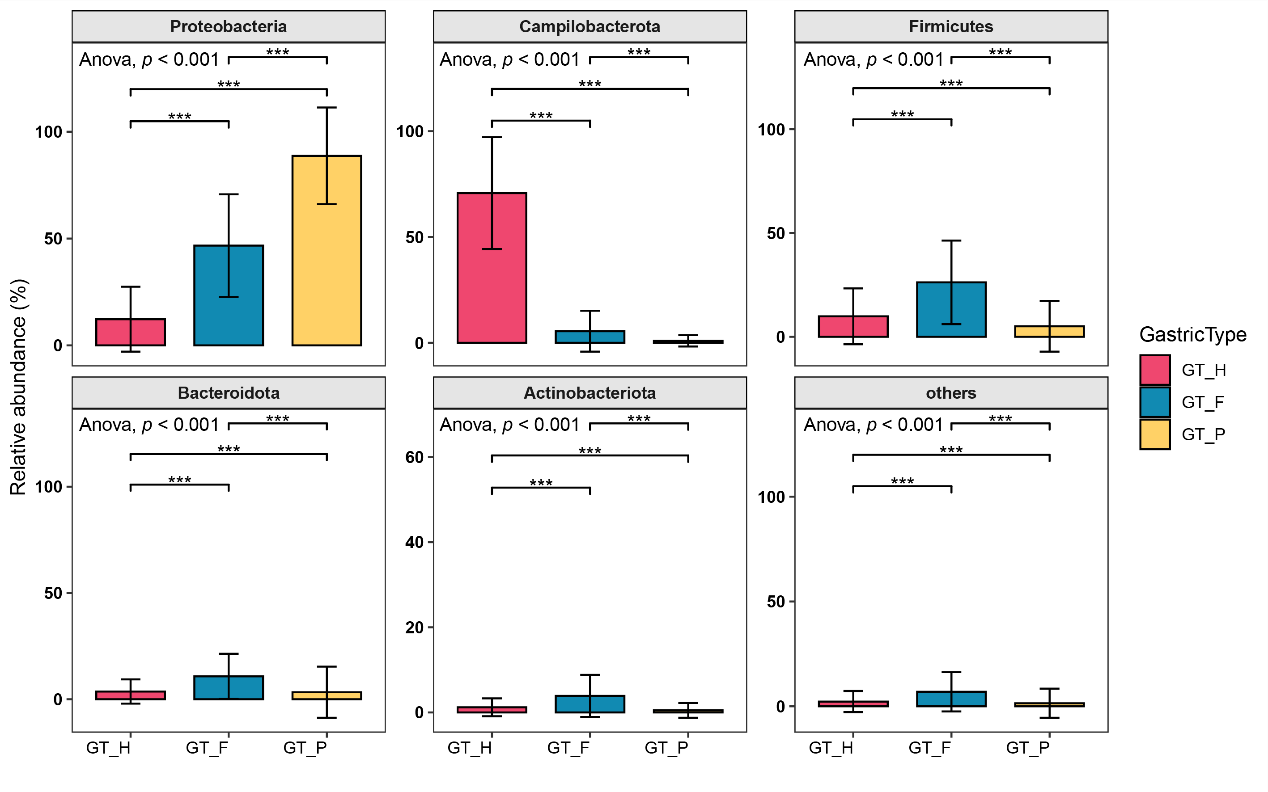


**Supplementary figure 3.** The distribution of the top abundant phylum in three microbiome communities. ANOVA test was used for comparison of bacterial relative abundance differences between multiple gastric microbial types, and multiple comparisons were performed by Tukey test and *p* values were adjusted. *: *p.adj <* 0.05, **: *p.adj <* 0.01, ***: *p.adj <* 0.001.


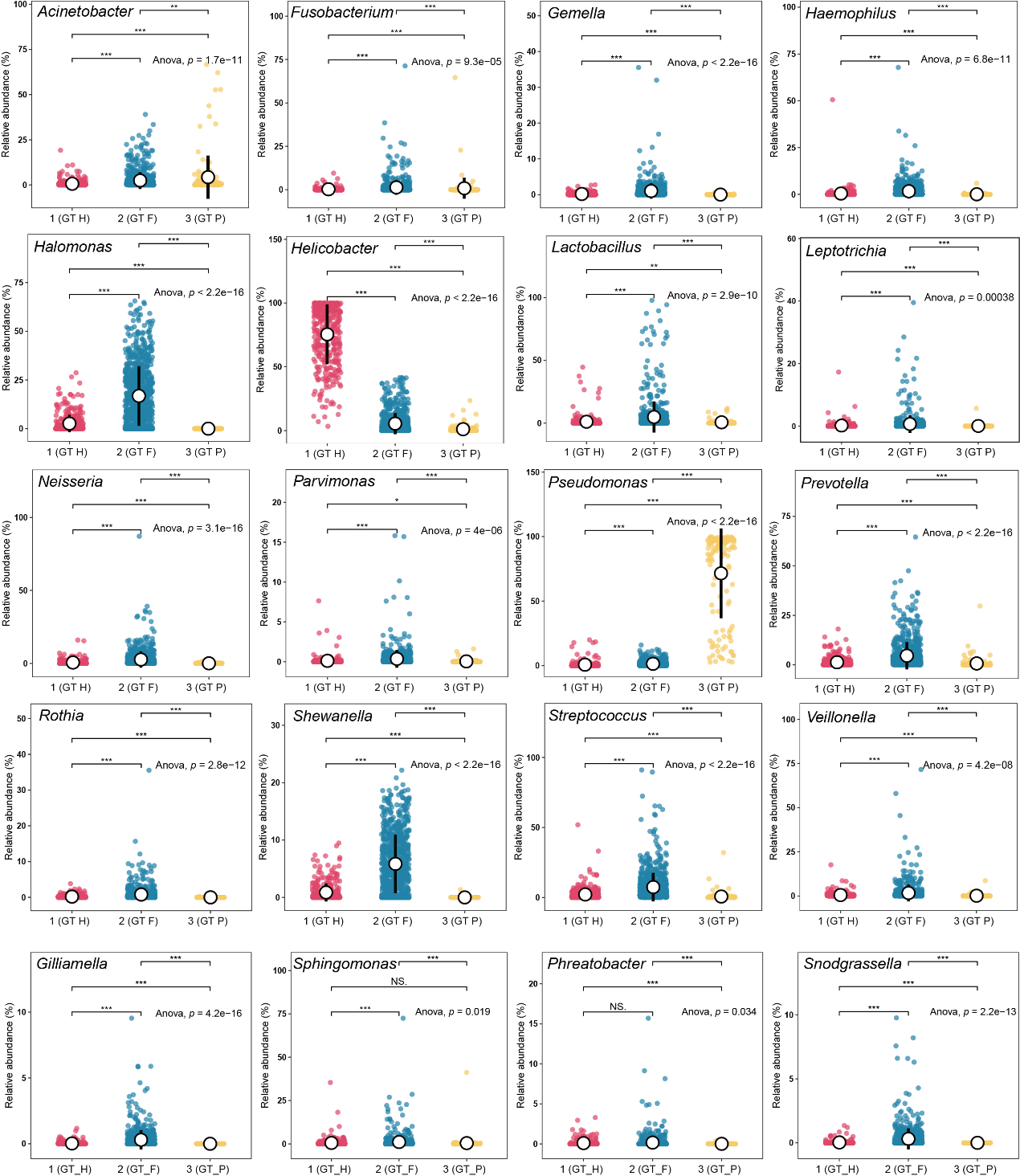


**Supplementary figure 4.** The distribution of the GC-associated bacteria in three microbiome communities. ANOVA test was used for comparison of bacterial relative abundance differences between multiple gastric microbial types, and multiple comparisons were performed by Tukey test and *p* values were adjusted. ** *p.adj <* 0.01, *** *p.adj <* 0.001.


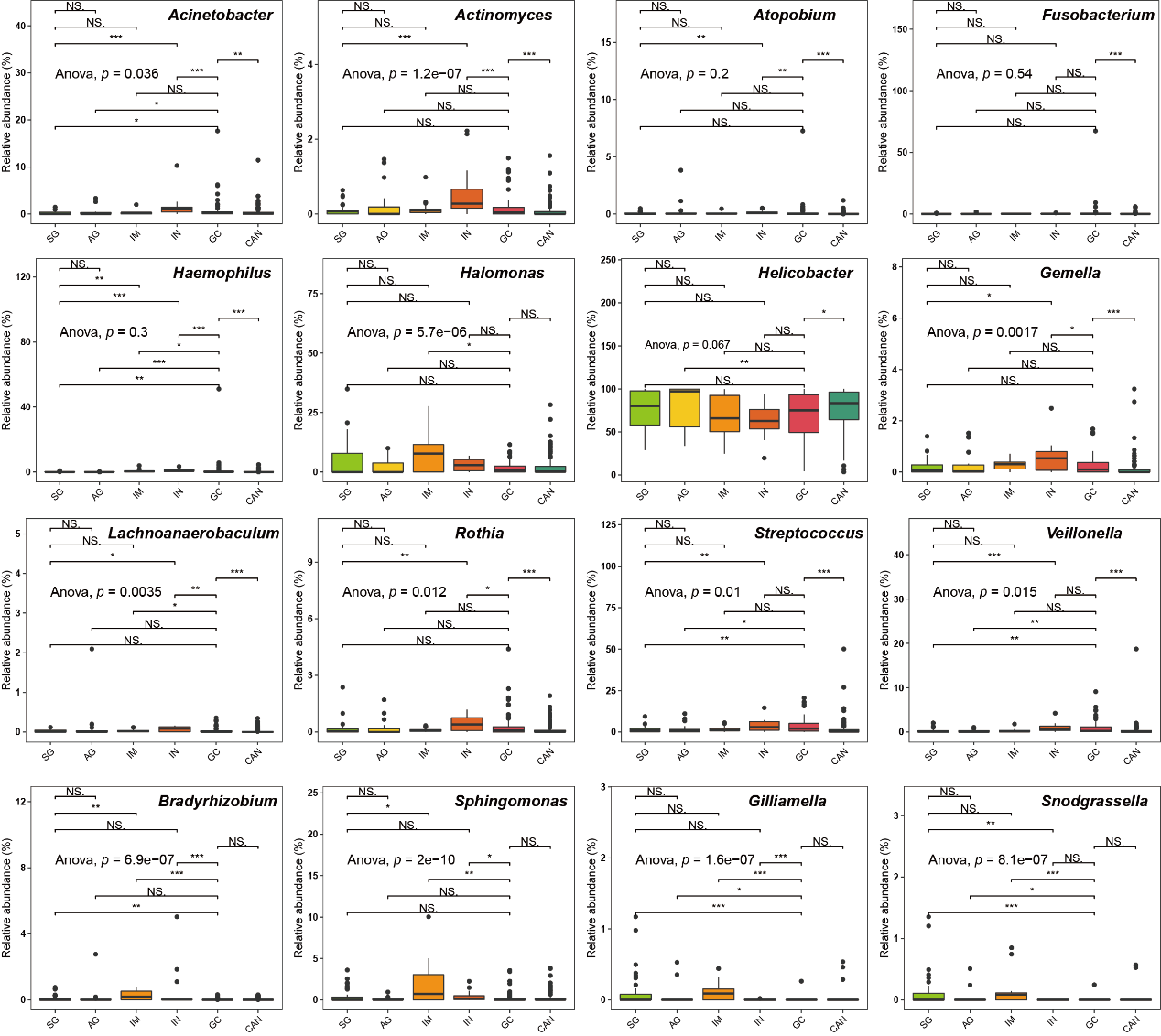


**Supplementary figure 5.** The distribution of the GC-associated bacteria in GT H type samples among different disease groups. ANOVA test was used for comparison of bacterial relative abundance differences between multiple gastric microbial types, and multiple comparisons were performed by Tukey test and *p* values were adjusted. * *p.adj* < 0.05, ** *p.adj* < 0.01, *** *p.adj* < 0.001.


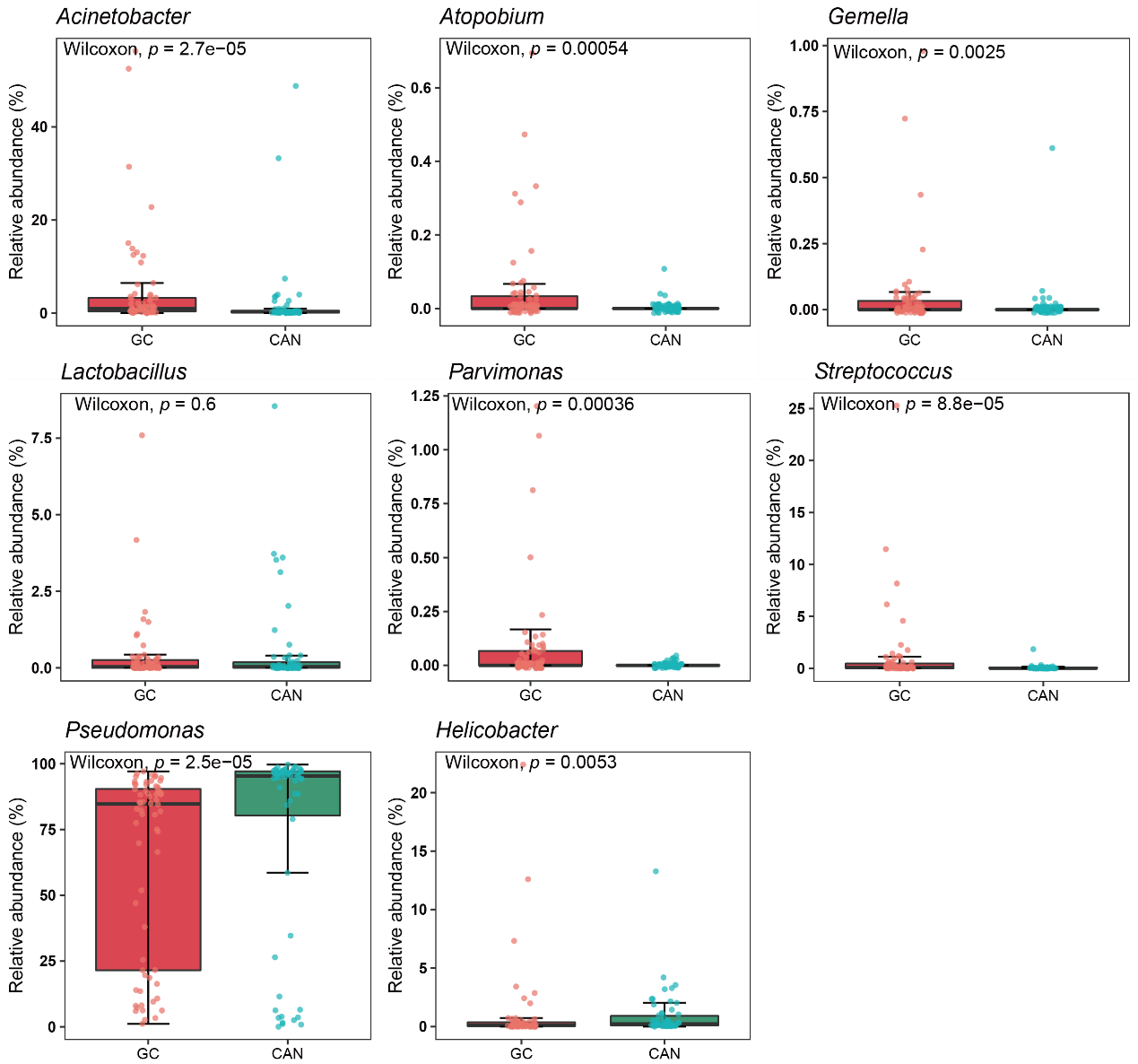


**Supplementary figure 6.** The distribution of the GC-associated bacteria in GT P type samples among different disease groups. Wilcoxon test was used for comparison of bacterial relative abundance differences between GC and CAN.


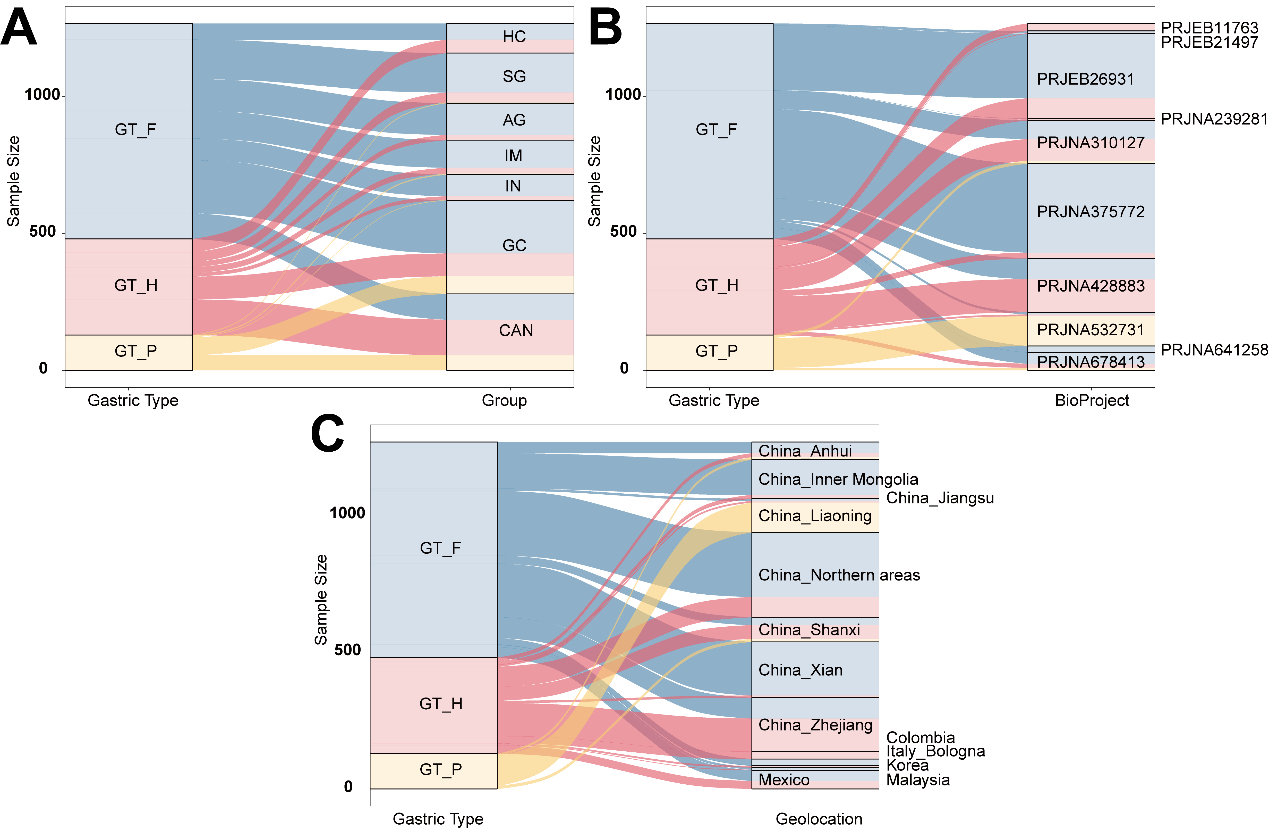


**Supplementary figure 7.** The distribution of samples with different gastric bacterial communities. (A) The distribution of different microbial type samples in multiple diagnosis groups. (B) The distribution of different microbial type samples in different datasets. (C) The distribution of different microbial type samples in different geolocations.


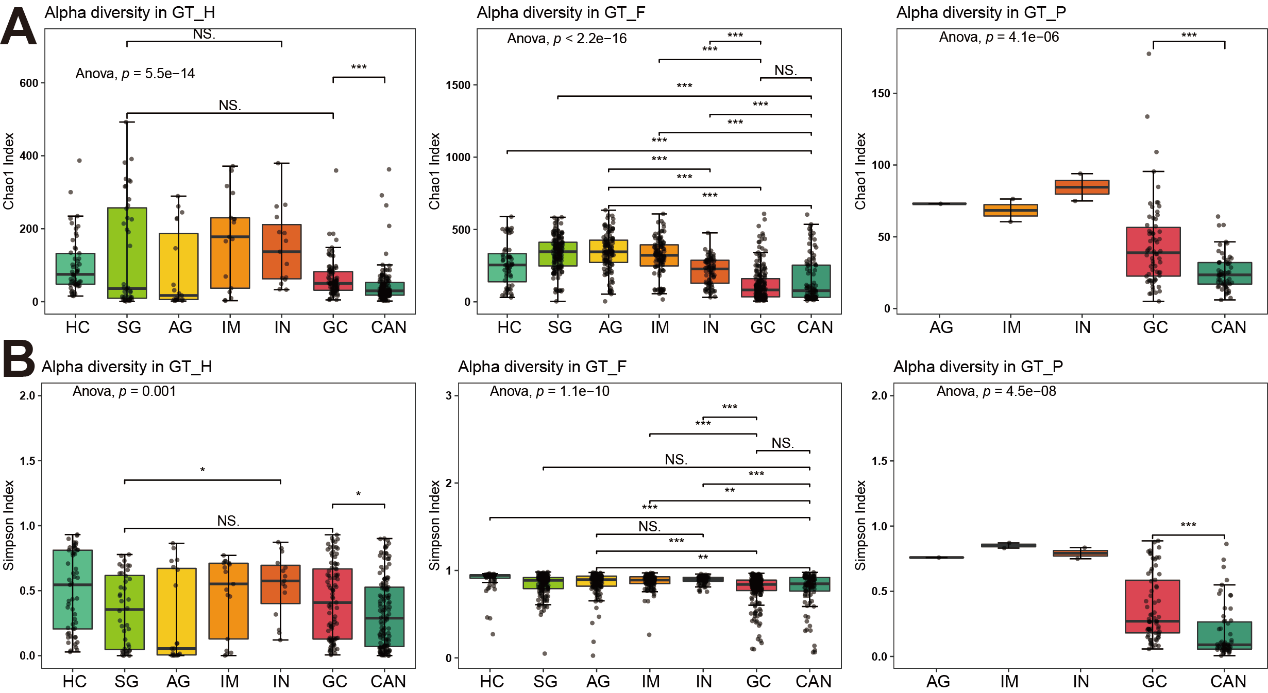


**Supplementary figure 8.** Gastric microbial diversity at the genus level among disease groups. (A) Alpha diversity was estimated by Chao1 richness diversity index for disease groups. (B) Alpha diversity was estimated by Simpson evenness index for disease groups. The ANOVA test was used for comparison of differences between multiple diagnostic groups. *: *p.adj <* 0.05, **: *p.adj <* 0.01, ***: *p.adj <* 0.001.


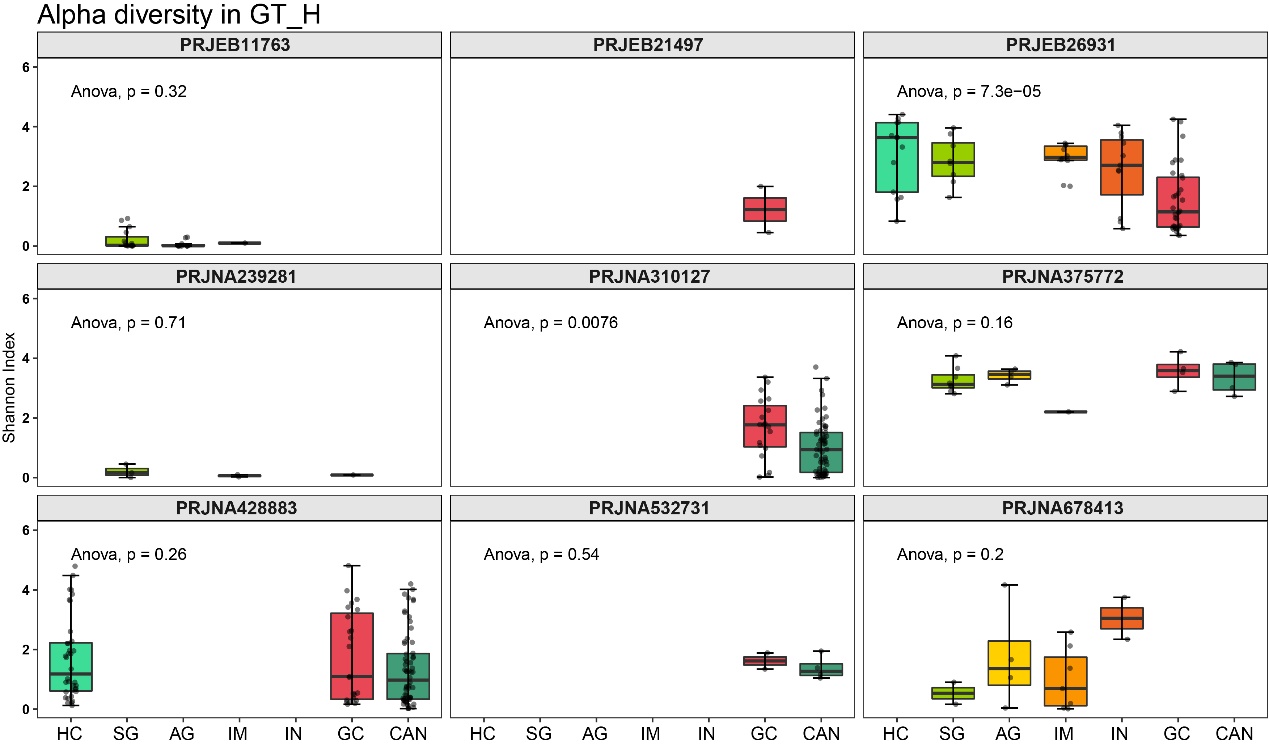


**Supplementary figure 9.** Gastric microbial α diversity of GT H type bacterial community among disease groups in different datasets. The ANOVA test was used for comparison of differences between multiple diagnostic groups.


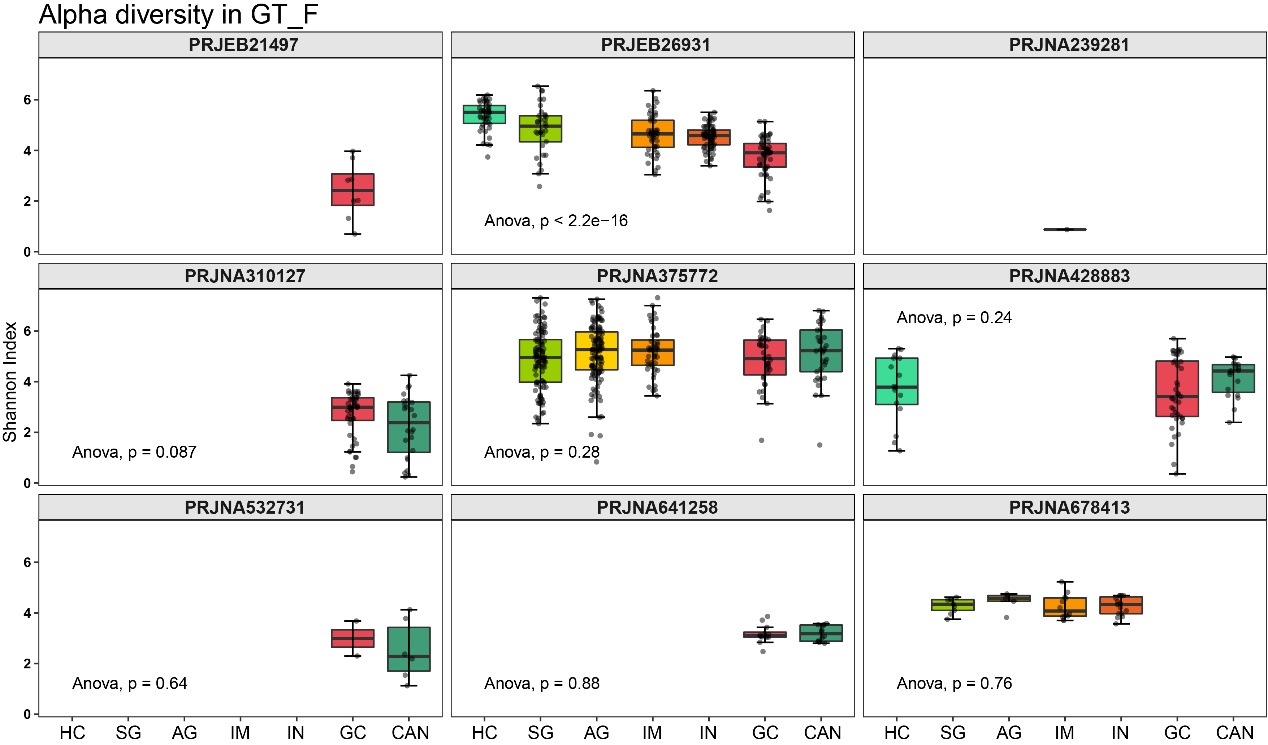


**Supplementary figure 10** Gastric microbial α diversity of GT F type bacterial community among disease groups in different datasets. The ANOVA test was used for comparison of differences between multiple diagnostic groups.


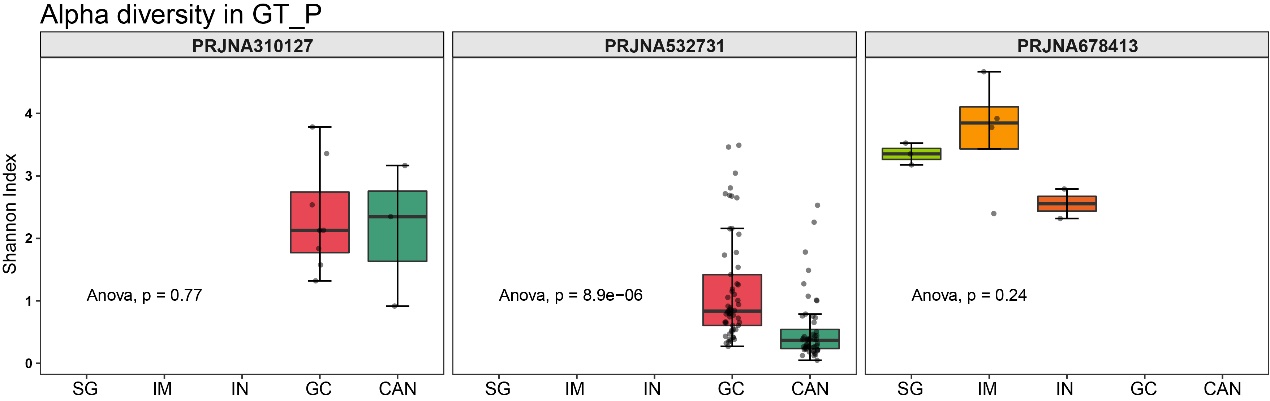


**Supplementary figure 11.** Gastric microbial α diversity of GT P type bacterial community among disease groups in different datasets. The ANOVA test was used for comparison of differences between multiple diagnostic groups.


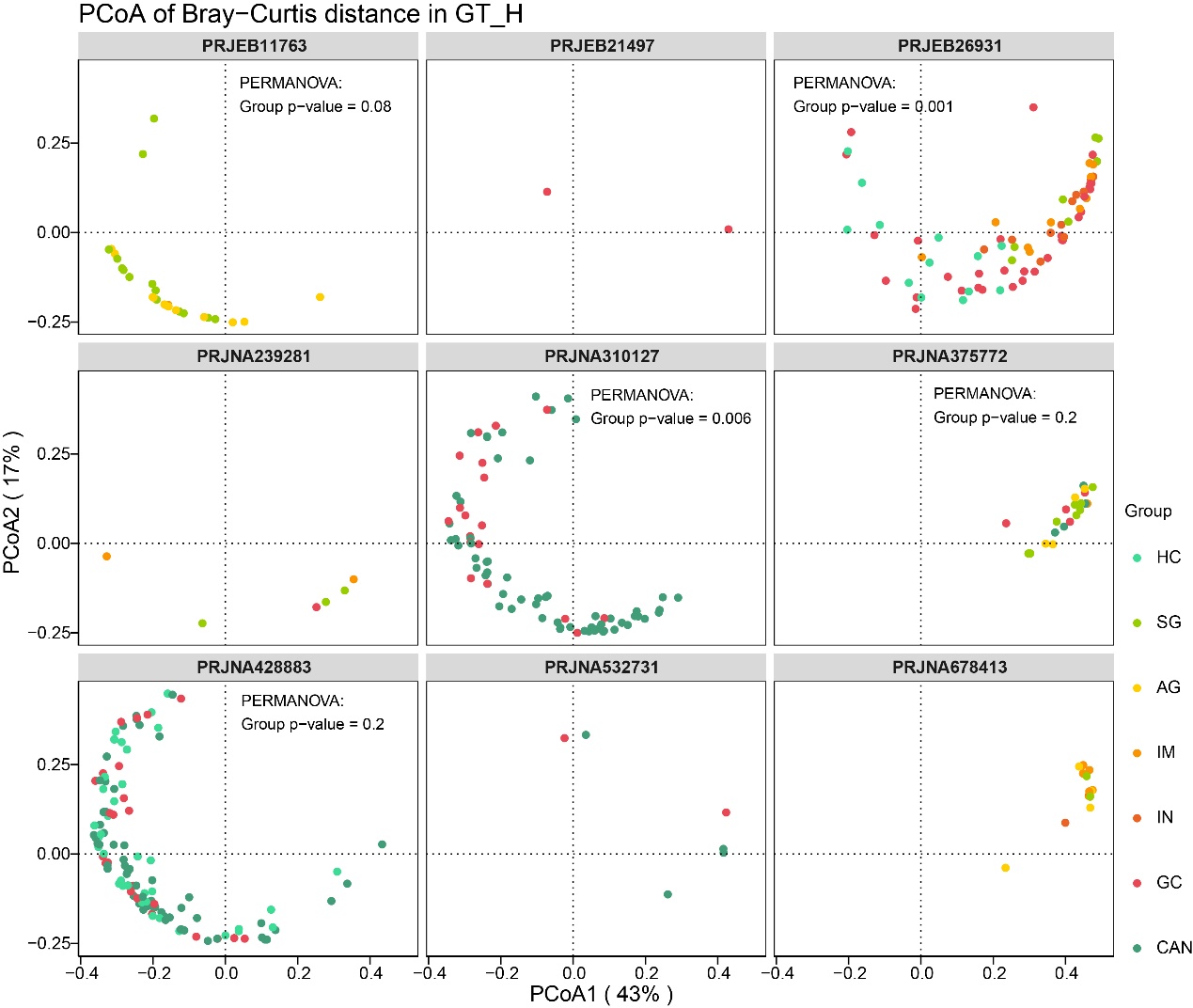


**Supplementary figure 12.** Gastric microbial β diversity of GT H type bacterial community among disease groups in different datasets. The PERMANOVA test was used for comparison of differences between multiple diagnostic groups.


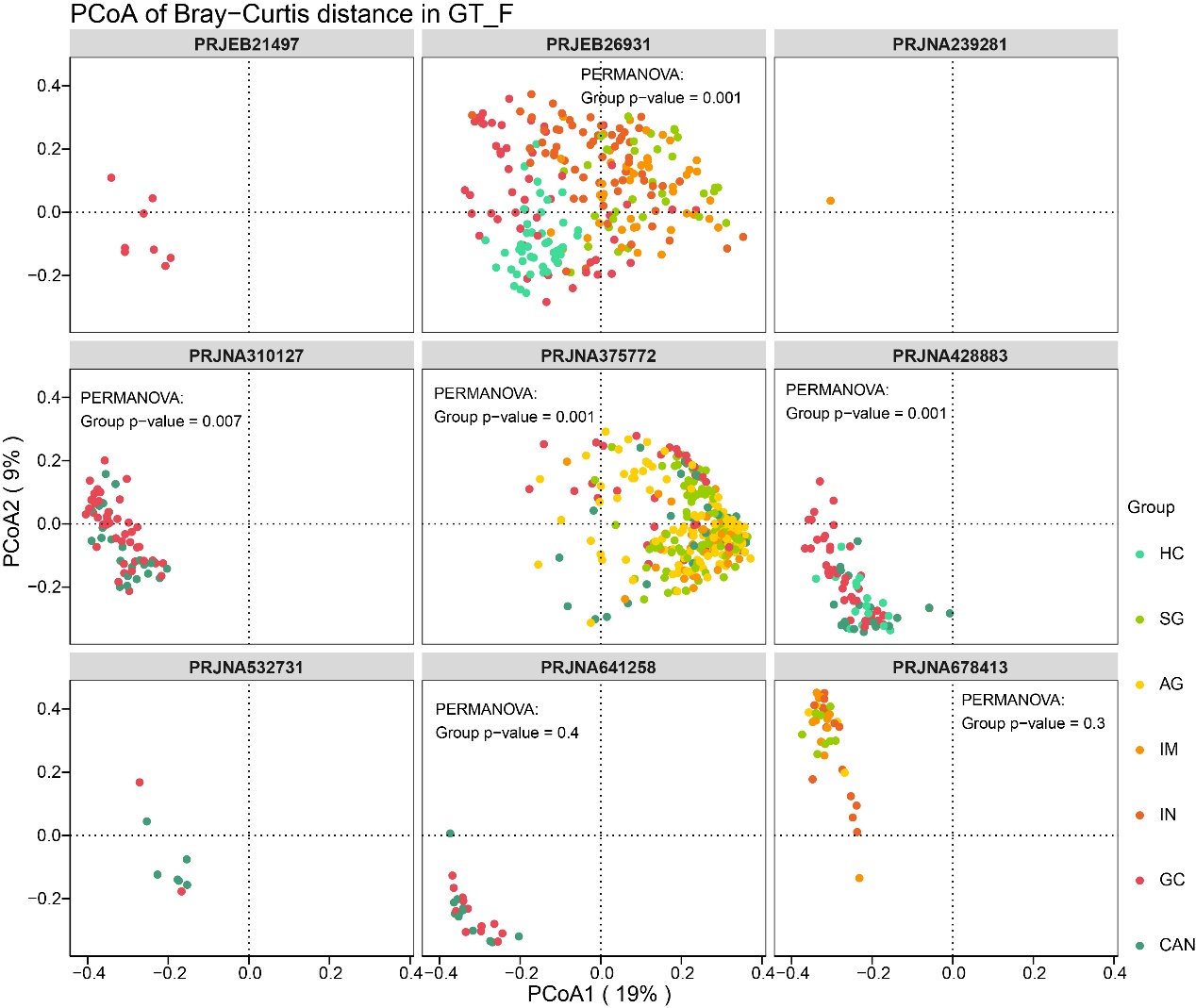


**Supplementary figure 13.** Gastric microbial β diversity of GT F type bacterial community among disease groups in different datasets. The PERMANOVA test was used for comparison of differences between multiple diagnostic groups.


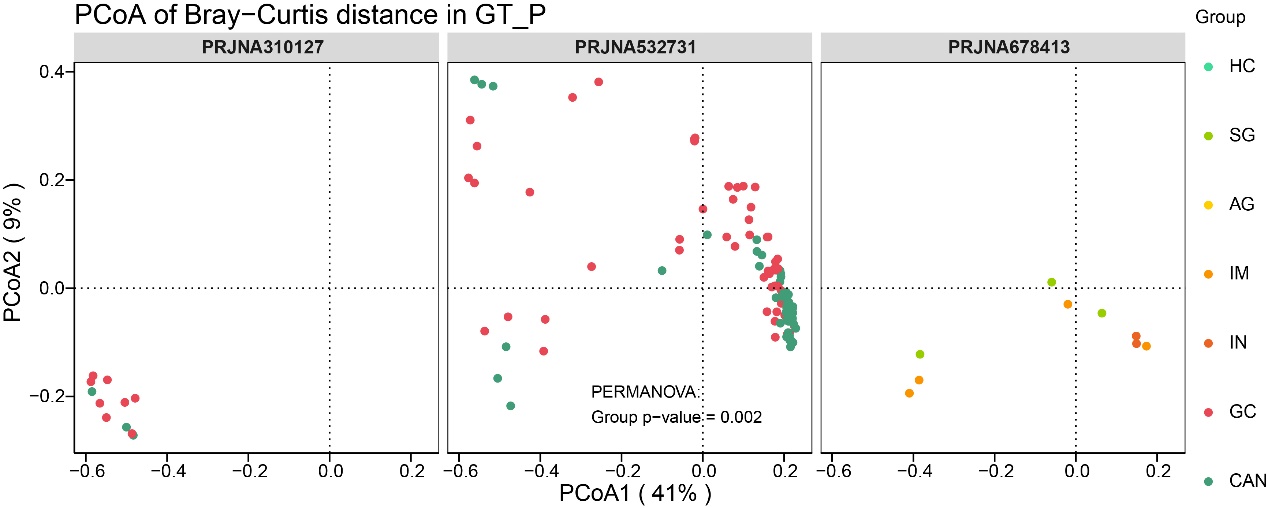


**Supplementary figure 14.** Gastric microbial β diversity of GT P type bacterial community among disease groups in different datasets. The PERMANOVA test was used for comparison of differences between multiple diagnostic groups.


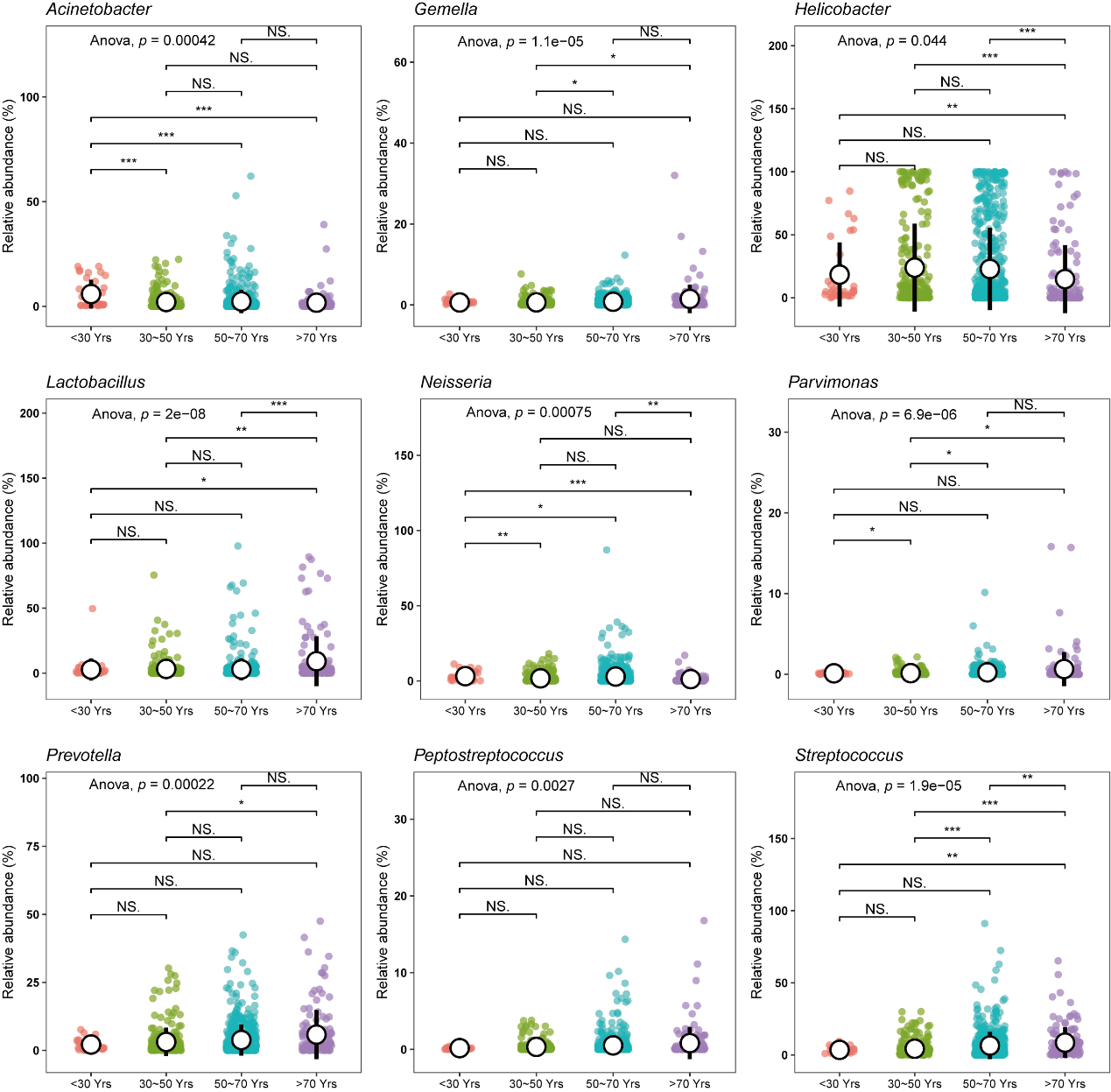


**Supplementary figure 15.** The distribution of the GC-associated bacteria in different periods of age. The ANOVA test was used for comparison of differences between multiple groups, and multiple comparisons were performed by Tukey test and *p* values were adjusted. * *p.adj <* 0.05, ** *p.adj <* 0.01, *** *p.adj <* 0.001.


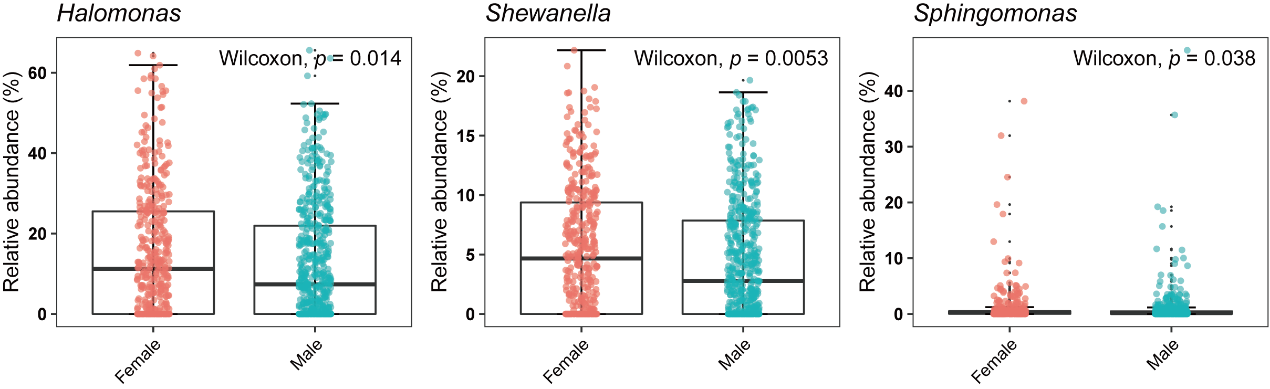


**Supplementary figure 16.** The distribution of the GC-associated bacteria in different sexes. The Wilcoxon test was used for comparison of differences between groups.


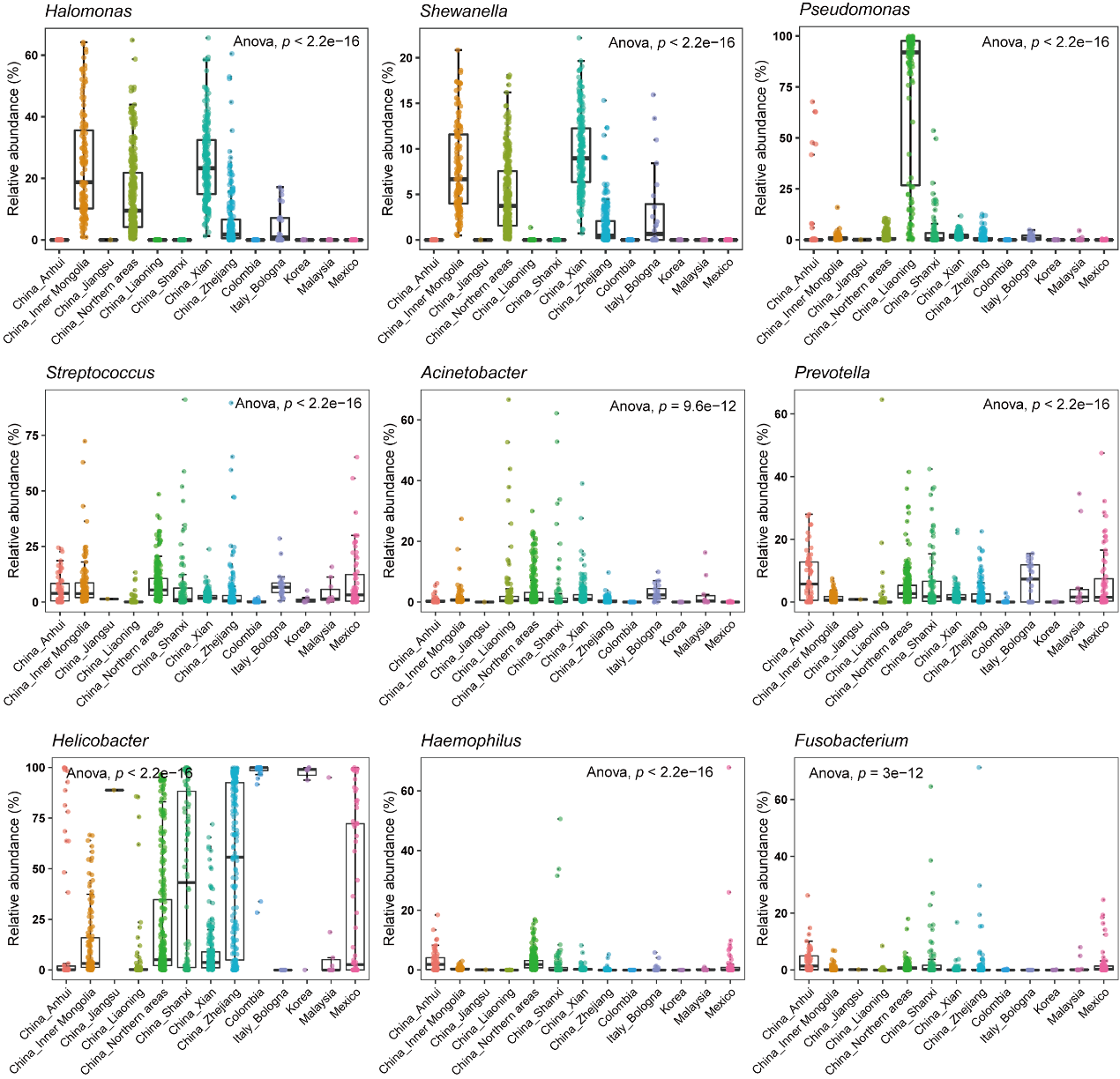


**Supplementary figure 17.** The distribution of the GC-associated bacteria in different geographical locations. The ANOVA test was used for comparison of bacterial relative abundance differences between multiple groups.


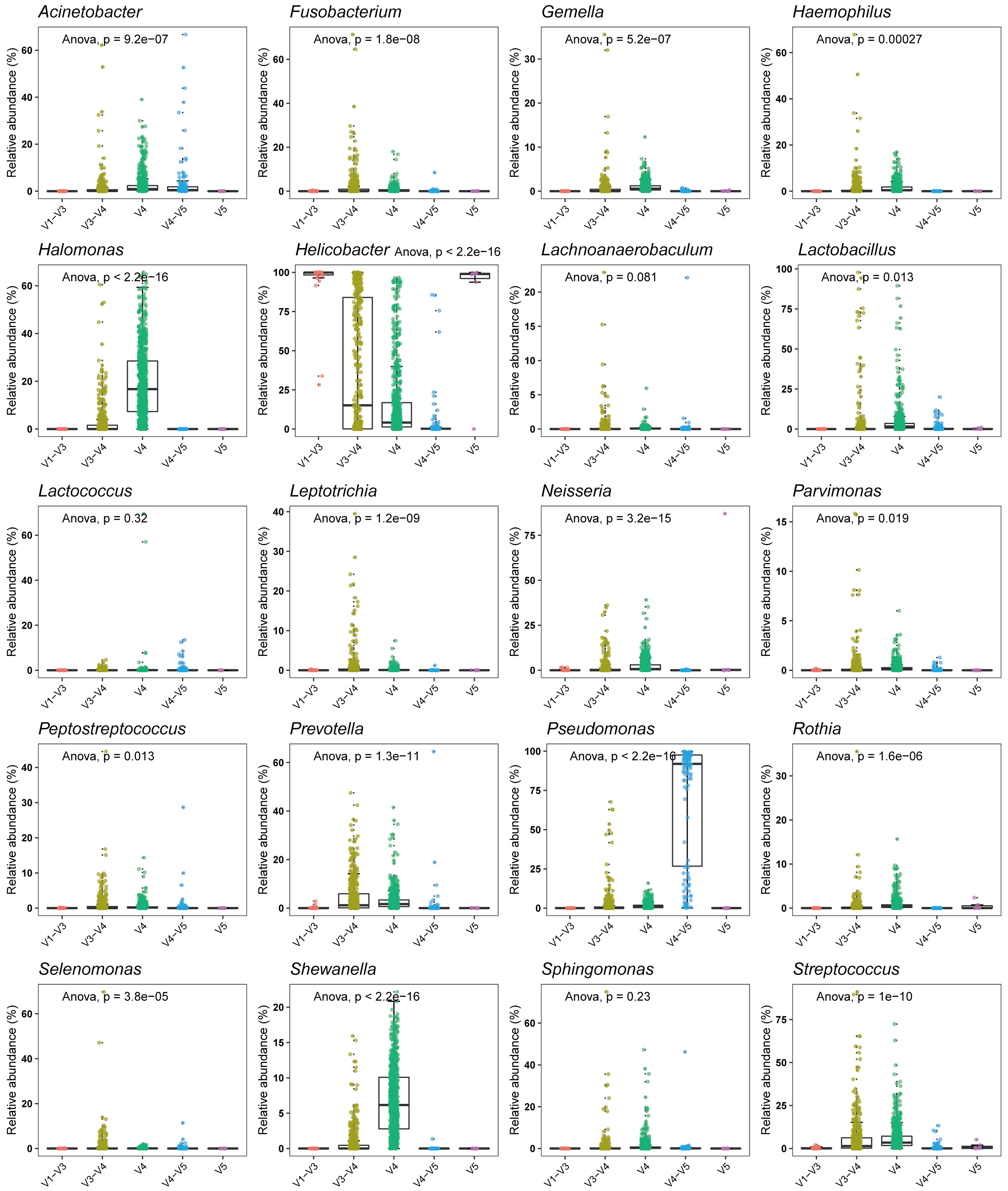


**Supplementary figure 18.** The distribution of the GC-associated bacteria in datasets with different sequencing region. The ANOVA test was used for comparison of bacterial relative abundance differences between multiple groups.


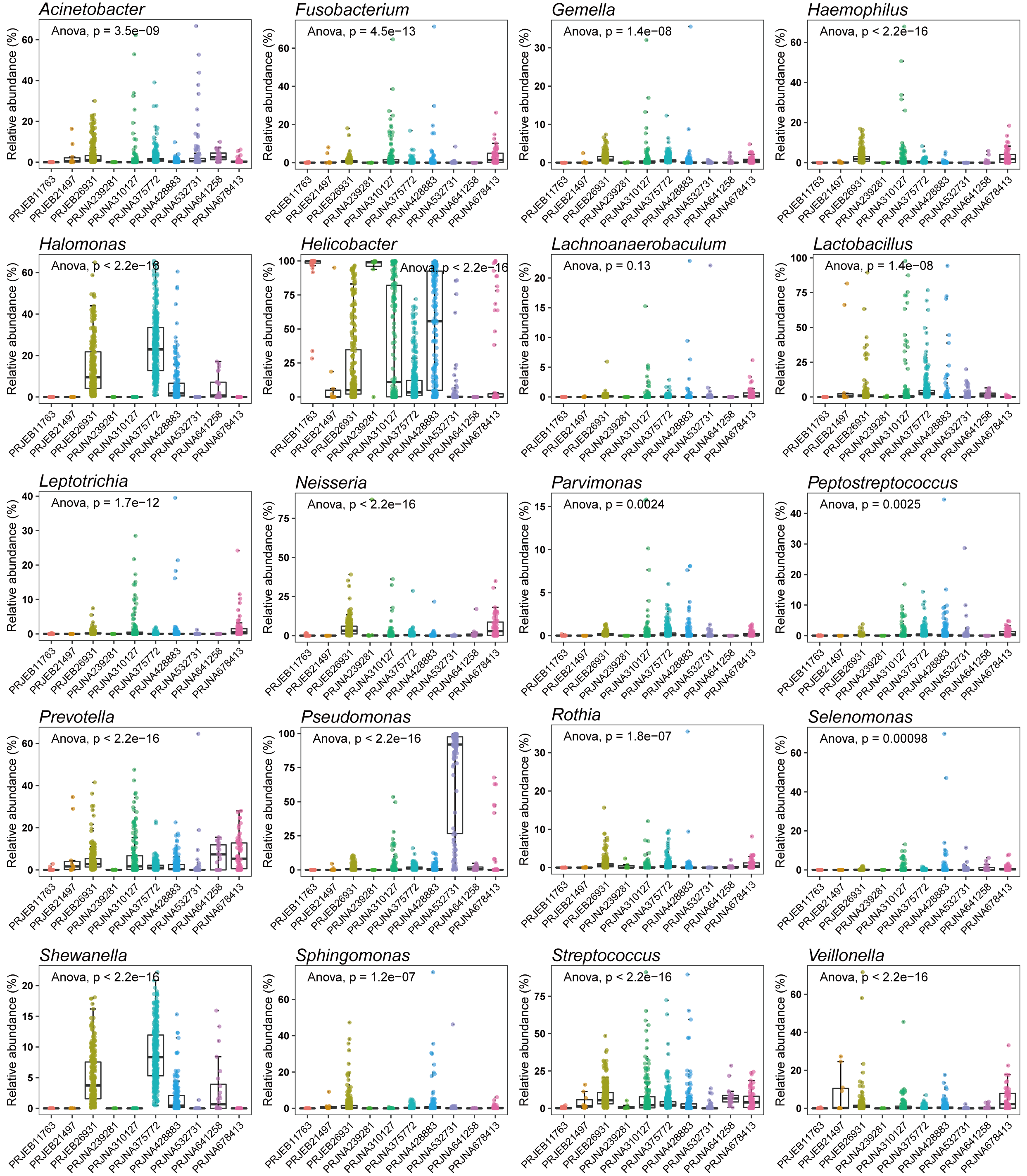


**Supplementary figure 19.** The distribution of the GC-associated bacteria in different datasets. The ANOVA test was used for comparison of bacterial relative abundance differences between multiple groups.
